# Endothelial Brg1 fine-tunes Notch signaling during zebrafish heart regeneration

**DOI:** 10.1038/s41536-023-00293-4

**Published:** 2023-04-07

**Authors:** Chenglu Xiao, Junjie Hou, Fang Wang, Yabing Song, Jiyuan Zheng, Lingfei Luo, Jianbin Wang, Wanqiu Ding, Xiaojun Zhu, Jing-Wei Xiong

**Affiliations:** 1 Beijing Key Laboratory of Cardiometabolic Molecular Medicine, Institute of Molecular Medicine, College of Future Technology, and State Key Laboratory of Natural and Biomimetic Drugs, Peking University, 100871 Beijing, China; 2 National Key Laboratory of Veterinary Public Health Security, College of Veterinary Medicine, China Agricultural University, 100193 Beijing, China; 3 College of Pharmaceutical Science, Zhejiang University of Technology, 310014 Hangzhou, China; 4 School of Life Sciences, Tsinghua University, 100084 Beijing, China; 5 Institute of Developmental Biology and Regenerative Medicine, Southwest University, Beibei, 400715 Chongqing, China

**Keywords:** Regeneration, Epigenetics

## Abstract

Myocardial Brg1 is essential for heart regeneration in zebrafish, but it remains unknown whether and how endothelial Brg1 plays a role in heart regeneration. Here, we found that both *brg1* mRNA and protein were induced in cardiac endothelial cells after ventricular resection and endothelium-specific overexpression of dominant-negative *Xenopus* Brg1 (*dn-xbrg1*) inhibited myocardial proliferation and heart regeneration and increased cardiac fibrosis. RNA-seq and ChIP-seq analysis revealed that endothelium-specific overexpression of *dn-xbrg1* changed the levels of H3K4me3 modifications in the promoter regions of the zebrafish genome and induced abnormal activation of Notch family genes upon injury. Mechanistically, Brg1 interacted with lysine demethylase 7aa (Kdm7aa) to fine-tune the level of H3K4me3 within the promoter regions of Notch family genes and thus regulated *notch* gene transcription. Together, this work demonstrates that the Brg1-Kdm7aa-Notch axis in cardiac endothelial cells, including the endocardium, regulates myocardial proliferation and regeneration via modulating the H3K4me3 of the *notch* promoters in zebrafish.

## Introduction

The high mortality and morbidity of myocardial infarction are of public concern worldwide. The loss of cardiomyocytes following myocardial infarction and the inadequate self-repair capability of the mammalian heart make it difficult to treat cardiac diseases^1^. As one of the least regenerative organs in the human body, the heart replaces the infarcted myocardium with a noncontractile scar instead of new muscles, which is initially beneficial but eventually leads to loss of contraction and function. Although various cell-based and cell-free strategies have been explored to restore infarcted heart function, the efficacy and side effects, such as arrhythmia and immune rejection, currently prevent translation to the clinic. The neonatal mouse can regenerate its heart, but this ability is lost after 7 postnatal days^2–4^. A number of elegant studies have provided evidence for the underlying mechanisms, but how to efficiently stimulate mammalian heart regeneration remains largely unknown. Unlike mammals, some lower vertebrates, such as zebrafish, can fully regenerate the heart after injury throughout life^5^. Dissecting the cellular and molecular mechanisms of zebrafish heart regeneration may provide clues for promoting heart regeneration in mammals.

It is conceivable that cardiomyocyte dedifferentiation and proliferation contribute to heart regeneration in zebrafish^6,7^. Over the past decades, a number of signaling pathways and transcription factors have been reported to regulate myocardial proliferation and regeneration in zebrafish, including fibroblast growth factor, sonic hedgehog, retinoic acid, insulin-like growth factor, Notch, GATA4, Hand2, NF-kB, and Stat3^8–13^. Retinaldehyde dehydrogenase 2, which produces retinoic acid, is activated in the epicardium and endocardium within hours after injury, and transgenic inhibition of retinoic acid receptors impairs myocardial proliferation^8^. Conditional inhibition of Notch signaling via overexpression of dominant-negative Notch transcriptional co-activator Master-mind like-1 (MAML) in endothelial cells (including the endocardium) decreases myocardial proliferation^12,14^. These studies suggest an essential role of endocardial signaling in regulating myocardial proliferation, but it remains to be addressed how endocardial Notch components are regulated or how endocardial signals regulate myocardial proliferation and regeneration upon injury.

Epigenetic regulation plays an important role in gene expression in various cellular processes such as differentiation, proliferation, fate determination, as well as organ regeneration^15–18^. Epigenetic regulation is, in general, defined as controlling gene expression beyond the DNA sequence itself, consisting of histone modifications, DNA/RNA modifications, noncoding RNAs, and chromatin-remodeling complexes^19^. The SWI/SNF (SWItch/Sucrose Non-Fermentable)-like complex, a member of the ATP-dependent chromatin-remodeling complex family, uses energy from ATP hydrolysis, regulates gene transcription by rearranging nucleosome positions and histone-DNA interactions, and thus facilitates the transcriptional activation or repression of targeted genes^20^. We previously reported that its central subunit, brahma-related gene 1 (*brg1* or *smarca4*), had a critical function in zebrafish heart regeneration by interacting with DNA (cytosine-5)-methyltransferase 3 alpha b to modify DNA methylation of the cyclin-dependent kinase inhibitor 1C promoter^21^. We found that *brg1* was not only induced in cardiomyocytes but also in cardiac endothelial cells, including the endocardium, during myocardial regeneration^21^. In this work, we investigated how endothelial Brg1 played a role in zebrafish heart regeneration. Inhibition of Brg1 via dominant-negative (DN)-xBrg1 in cardiac endothelial/endocardial cells decreased myocardial proliferation and heart regeneration, and Brg1 interacted with the histone demethylase Kdm7aa (lysine (K)-specific demethylase 7Aa) to regulate *notch* receptor gene expression upon injury. Together, this work supports the notion that the Brg1-Kdm7aa axis fine-tunes Notch signaling in cardiac endothelium and endocardium during heart regeneration.

## Results

### Endothelial Brg1 is required for heart regeneration in zebrafish

**Fig. 1:**
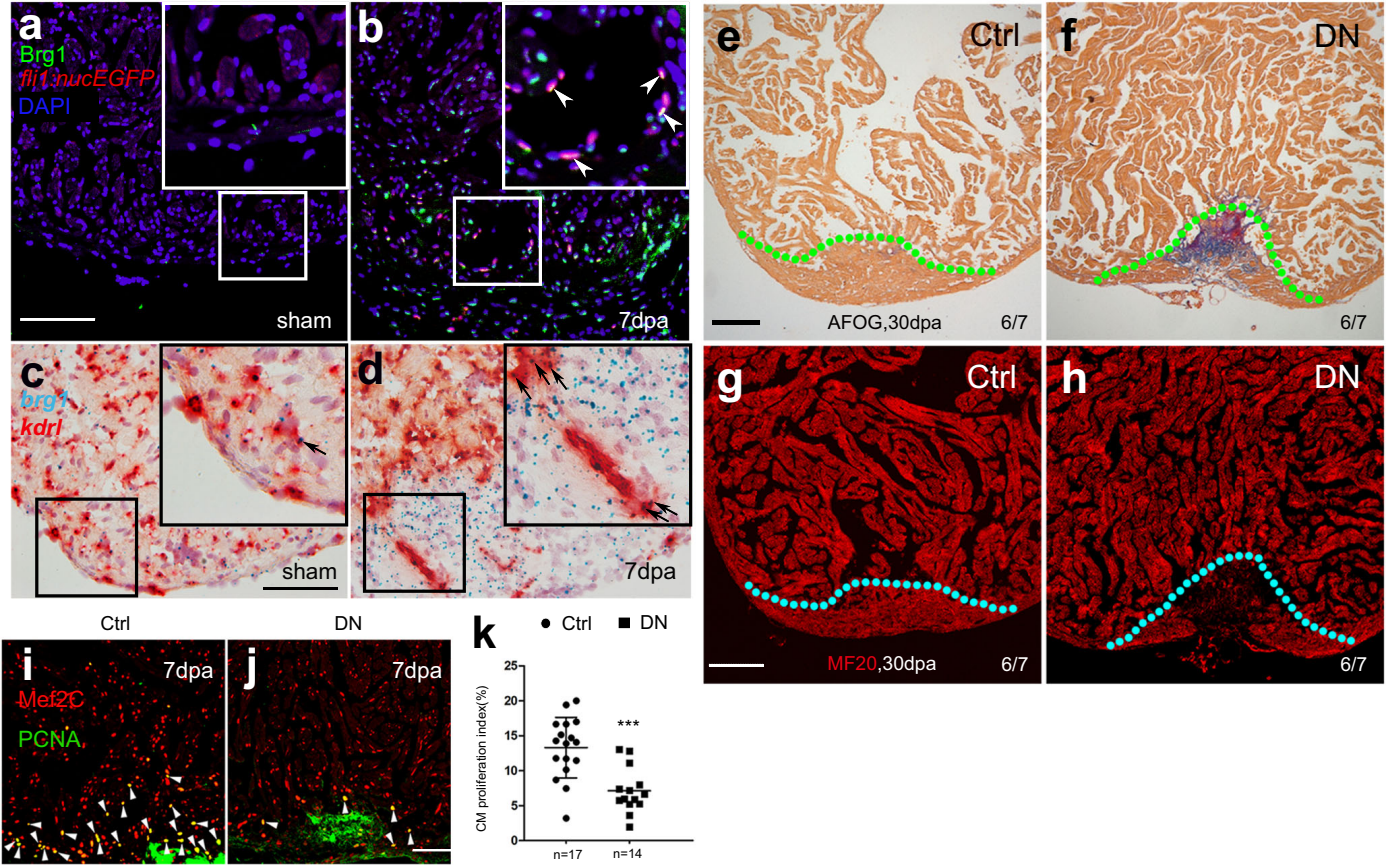
Inhibition of endothelial Brg1 impairs myocardial proliferation and regeneration. **a**, **b** Immunofluorescence staining of Brg1 and EGFP on paraffin sections of *Tg(fli1:nucEGFP)* transgenic hearts from sham-operated (**a**) and injured zebrafish hearts (**b**) at 7 dpa (arrowheads, Brg1- and EGFP-positive endothelial cell nuclei). **c**, **d** RNAscope in situ hybridization of *brg1* and *kdrl* probes in frozen sections from sham-operated (**c**) and injured hearts (**d**) at 3 dpa (arrows, *brg1*- and *kdrl*-positive endothelial cells). The upper right corner presented in (**a**–**d**) is a high-magnification image of the framed area. **e**–**h** Representative images of Acid fuchsin orange G (AFOG) staining (**e**, **f**) and immunofluorescence with anti-myosin heavy chain (MF20) (**g**, **h**) of heart sections from control siblings *Tg(ubi:LoxP-DsRed-STOP-LoxP-dn-xbrg1)* (Ctrl) and endothelium-specific dominant-negative *brg1* mutants *Tg(ubi:LoxP-DsRed-STOP-LoxP-dn-xbrg1; kdrl:CreER)* (DN) at 30 dpa, noting that, compared with robust regenerated myocardium and rare cardiac fibrosis in Ctrl group (**e**, **g**), the DN group failed to regenerate the myocardium (**h**) and had evident fibrin (red) and collagen (blue) deposition (**f**). Dashed lines mark the resection traces. N numbers indicate biological replicates. **i**, **j** Immunostaining of representative heart sections at 7 dpa identified cardiomyocyte nuclei (Mef2C^+^) and nuclei undergoing DNA replication (PCNA^+^). Noting fewer proliferative cardiomyocytes (Mef2C^+^/PCNA^+^) in the DN group than in the Ctrl group. Arrowheads, Mef2C^+^/PCNA^+^ proliferating cardiomyocytes. **k** Statistical analysis of experiments as in (**i**) and (**j**) (CM, cardiomyocyte; *n* = 17 or 14 biological replications; data were the mean percentage ± s.e.m.; ***p < 0.001, unpaired *t*-test). Scale bars, 100 μm.

Our previous work has shown that both global and myocardium-specific inhibition of Brg1 results in impaired myocardial proliferation and regeneration, while global inhibition of Brg1 leads to more severe cardiac fibrosis than its myocardium-specific inhibition^21^. In addition to elevated expression in the injured myocardium, Brg1 was also induced in other cardiac cells, including endothelial cells, during heart regeneration. To evaluate Brg1 expression in endothelial cells during zebrafish heart regeneration, we used immunofluorescence staining (Fig. 1a, b) and RNAscope in situ hybridization (Fig. 1c, d) to determine whether Brg1 was induced in endothelial cells upon ventricular amputation. Consistent with our previous report, Brg1 protein was co-localized with *Tg(fli1:nucEGFP)*-positive endothelial cells in the injury site at 7 days post-amputation (dpa) (Fig. 1a, b). Moreover, RNAscope staining revealed that *brg1* mRNA was elevated and partially overlapped with *kdrl*-positive endothelium at 3 dpa (Fig. 1c, d). We then turned to tamoxifen-induced endothelium-specific inhibition of Brg1 with the transgenic strains *Tg(ubi:LoxP-DsRed-STOP-LoxP-dn-xbrg1; kdrl:CreER)*^21,22^ to address whether Brg1 had a function in endothelial cells during regeneration. To validate the endothelial-specific expression of *Tg(kdrl:CreER)*, we created compound transgenic zebrafish line *Tg(ubi:LoxP-DsRed-STOP-LoxP-EGFP; kdrl:CreER)*, where endothelial-specific expression of GFP is driven by the *kdrl* promoter after Cre-mediated recombination. The immunostaining with GFP antibody showed that GFP was expressed in the endocardium/coronary vessels in the double transgenic fish hearts treated with 4-HT (Supplementary Fig. 1a). Furthermore, RNA in situ analysis with either *CreER* or *dn-xbrg1* probes revealed expression of *dn-xbrg1* and *CreER* mRNA in the endocardium/coronary vessels on frozen heart sections from *Tg(ubi:LoxP-DsRed-STOP-LoxP-dn-xbrg1; kdrl:CreER)* [DN] hearts but not *Tg(ubi*:*LoxP-DsRed-STOP-LoxP-dn-xbrg1)* [Ctrl] hearts treated with tamoxifen (Supplementary Fig. 1b). Interestingly, endothelium-specific overexpression of *dn-xbrg1* resulted in abnormal cardiac fibrosis (Fig. 1e, f) and compromised myocardial regeneration (Fig. 1g, h) at 30 dpa as well as decreased the number of proliferating cardiomyocytes at 7 dpa (Fig. 1i–k). Using RNAscope in situ hybridization, we also found that endothelium-specific inhibition of Brg1 decreased the number of *kdrl*-positive endothelial cells (Supplementary Fig. 2a–c) and *coronin1a*-positive leukocytes (Supplementary Fig. 2d–f) while having no effect on *tcf21*-positive epicardium (Supplementary Fig. 2g–i) in DN hearts compared with Ctrl sibling hearts at 7 dpa in the presence of 4-hydroxytamoxifen (4-HT). Taken together, these data demonstrate that endothelial Brg1 is required for myocardial proliferation during heart regeneration.

### Endothelium-specific inhibition of Brg1 changes the levels of H3K4me3 in the promoter regions of zebrafish genome

**Fig. 2:**
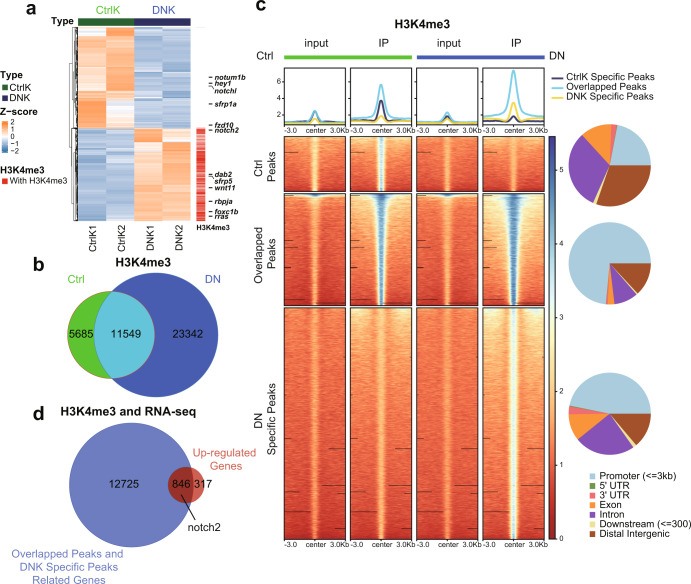
Endothelium-specific inhibition of Brg1 changes the levels of H3K4me3 in the promoter regions of the zebrafish genome. **a** Heatmap displaying *Z-*score normalized gene expression for differentially expressed genes between FACS-sorted *kdrl:EGFP* endothelial cells from dominant-negative Brg1 groups (DNK1 and DNK2) and control groups (CtrlK1 and CtrlK2). FPKM value of each gene was normalized using *Z*-scores across samples. Columns represent individual samples (two biological replicates for each group); rows represent differentially expressed genes ordered by hierarchical clustering. Labeled genes are part of the differentially expressed Notch signaling genes. The upregulated genes in the DNK group that were labeled with ‘red color’ had H3K4me3 peaks in their promoters. **b** Venn plot representing the intersection of H3K4me3 peaks between Ctrl and DN groups. **c** Heatmaps and summary plots displaying the signal profile of normalized read coverage around three categories of H3K4me3 peaks across different samples. The read coverage was normalized to 1x sequencing depth in all samples. Each row of the heatmap represents one peak, with coverage plotted across the 3 kb surrounding the peak summit. H3K4me3 peaks were classified into three categories: Ctrl-Specific Peaks represent peaks specifically in the Ctrl group; Overlapped Peaks represent peaks overlapped between the Ctrl and DN groups; DN-Specific Peaks represent peaks specifically in the DN group. The genomic distribution for three types of peaks is presented with pie charts on the right side. **d** Venn plot representing the intersection between genes with promoters marked by Overlapped Peaks and DN-Specific Peaks and genes that were differentially upregulated in the DNK group.

To decipher the molecular action of endothelial Brg1, we used RNA-seq analysis to search for Brg1-regulated genes during heart regeneration. We applied *Tg(kdrl:EGFP)* to label cardiac endothelial cells, including the endocardium, and achieved endothelium-specific overexpression of *dn-xbrg1* by using the compound zebrafish line consisting of *Tg(ubi:LoxP-DsRed-STOP-LoxP-dn-xbrg1; kdrl:CreER; kdrl:EGFP)* (defined as DNK), while we used *Tg(ubi:LoxP-DsRed-STOP-LoxP-dn-xbrg1; kdrl:EGFP)* as control (CtrlK) in the presence of 4-HT starting at 3 days before ventricular resection. The *kdrl:EGFP* endothelial cells, which were sorted by fluorescence-activated cell sorting (FACS) from CtrlK and DNK hearts at 7 dpa (Supplementary Fig. 3), were subjected to RNA-seq analysis, and differentially expressed genes were identified (Fig. 2a). Compared with CtrlK group, we found 1163 upregulated genes and 1266 downregulated genes in DNK group (Fig. 2a; Supplementary Table 3). Gene ontology (GO) enrichment analysis of these genes revealed that receptor activity-related genes were among the top-affected leads, in which the Notch signaling component *notch2* was strongly induced in the DNK group (Fig. 2a; Supplementary Fig. 4a, b; Supplementary Table 4). Some other biological processes, such as cell adhesion, response to wounding, extracellular matrix organization, cell migration, wound healing, and immune response, were also significantly affected after the inhibition of Brg1, specifically in endothelial cells (Supplementary Fig. 4a; Supplementary Table 4).

It is well recognized that Brg1 is involved in both gene activation and repression through interacting with epigenetic modifiers and influencing histone modifications at the targeted gene promoters^23^. And previous studies have established that the nucleosomes with histone H3 Lysine 4 trimethylation (H3K4me3) are mainly associated with the promoter regions of active transcription^24,25^. Therefore, we examine whether endothelial-specific overexpression of *dn-xbrg1* has an effect on the level of the histone marker H3K4me3 in the zebrafish genome. Genome-wide ChIP-seq analyses of Ctrl and DN amputated ventricles at 7 dpa using H3K4me3 antibody revealed that, in addition to 11,549 overlapping H3K4me3 peaks between the Ctrl and DN groups, more H3K4me3 peaks emerged in the DN group, suggesting that inhibition of Brg1 enhanced H3K4me3 modifications (Fig. 2b). Peaks were then divided into three categories according to the Venn plot, namely Ctrl-Specific Peaks in Ctrl group, Overlapped Peaks representing peaks overlapped between Ctrl and DN groups, and DN-Specific Peaks representing peaks specifically in DN group. Heatmaps and summary plots of H3K4me3 ChIP-seq signals in 3 kb surrounding the peak summits displayed slightly stronger Ctrl-Specific Peaks signals in the Ctrl group, while increased Overlapped Peaks signals and DNK-Specific Peaks signals in the DN group (Fig. 2c). Moreover, genomic distribution analysis for three categories of peaks revealed that peaks with increased signals in DN group were more concentrated in the promoter region than that with decreased peak signals (Fig. 2c), suggesting that endothelial Brg1 inhibition led to elevated levels of H3K4me3 in the promoter regions. Apart from basic biological processes, we also found that GO terms about “Notch signaling pathway” were highly enriched near the overlapped peaks between Ctrl and DN groups (Supplementary Fig. 5b), and GO terms about “chromatin remodeling” were enriched near both DN-Specific Peaks and Overlapped Peaks (Supplementary Fig. 5). We then examined the correlation of differentially expressed genes from RNA-seq and H3K4me3 modification levels. We analyzed the overlapping genes by comparing upregulated genes in the DNK group with the genes whose promoters were marked by Overlapped Peaks and DN-Specific Peaks (Fig. 2d), as well as comparing downregulated genes in the DNK group with the genes whose promoters were marked by Overlapped Peaks and Ctrl-Specific Peaks. Venn plot identified 846 of the 1163 upregulated genes in the DNK group, which consisting of receptor activity-related Notch signaling component *notch2* are occupied with Overlapped Peaks and DN-Specific H3K4me3 Peaks in the promoter regions (Fig. 2d, Supplementary Fig. 4c). These data suggest that endothelial-specific inhibition of Brg1 results in increased H3K4me3 modification levels in the promoter region of genes, which in turn leads to upregulation of genes expression, including *notch2*, in DN hearts.

### Endothelium-specific inhibition of Brg1 induces upregulation of Notch signaling by increasing the level of H3K4me3 in the promoters

**Fig. 3:**
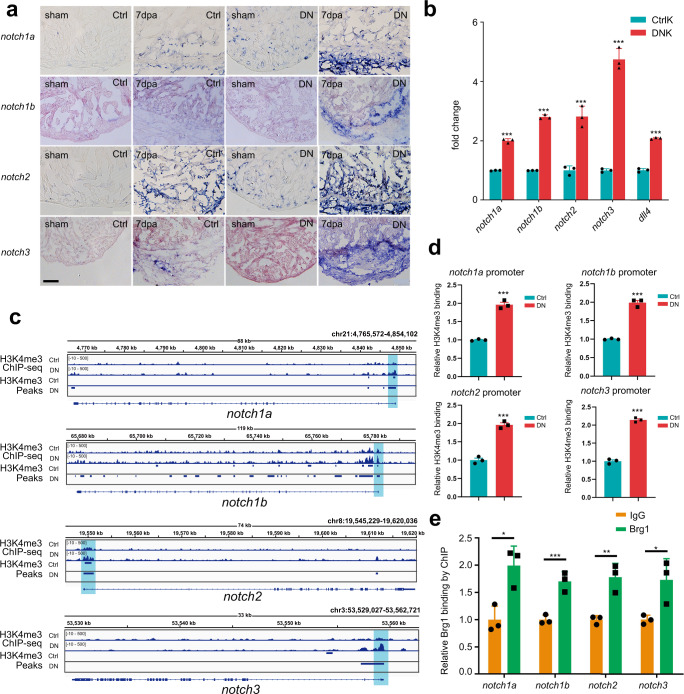
Endothelium-specific inhibition of Brg1 induces abnormal upregulation of Notch signaling via the increased levels of H3K4me3 in their promoters. **a** Representative images of RNA in situ hybridization with *notch1a*, *notch1b*, *notch2*, and *notch3* probes on frozen sections of sham-operated Ctrl hearts, injured Ctrl hearts, sham-operated DN hearts, and injured DN hearts at 7 dpa. Scale bar, 100 μm. **b** Quantitative RT-PCR analysis showing that the expression of *notch* receptors and ligand in FACS-sorted *kdrl:EGFP* endothelial cells from the DNK group was higher than those from the CtrlK group. Data represented one of three independent experiments. Data were mean fold changes ± s.e.m., ***p < 0.005, unpaired *t*-test. **c** H3K4me3 ChIP-seq showing the traces and peak intervals of representative genomic loci from Ctrl and DN hearts. H3K4me3 peaks in both Ctrl and DN groups were shown as bars. Putative promoter regions were indicated in blue color. **d** Anti-H3K4me3 ChIP and quantitative PCR in Ctrl and DN hearts at 7 dpa (primers designed from *notch* receptor genomic regions: *notch1a*, −171/+3 bp; *notch1b*, −41/+58 bp; *notch2*, −263/−115 bp; *notch3*, +394/+504 bp; ATG site designed as +1 bp). Data represented one of three independent experiments. Data were the mean fold changes ± s.e.m.; ***p < 0.005, unpaired *t*-test. **e** Anti-Brg1 ChIP and quantitative PCR in wild-type hearts at 3 dpa. Data represented one of two independent experiments. Data were the mean fold change ± s.e.m.; *p < 0.05, **p < 0.01, ***p < 0.005; unpaired *t*-test. *n* number shown here (**b**, **d**, **e**) indicated technical replicates.

Previous works by Zhao and colleagues have found that endocardial expression of all four *notch* receptors was induced after injury, while hyperactivation of Notch signaling blocked zebrafish heart regeneration^11^. However, how *notch* receptors were transcriptionally regulated during zebrafish heart regeneration is yet unknown. Importantly, from the above RNA- and ChIP-seq data, we found overexpression of *dn-xbrg1* increased the levels of H3K4me3 modifications and mRNA expression of *notch2*, and GO terms about “Notch signaling pathway” were highly enriched near the overlapped peaks between Ctrl and DN group, we then ask how Brg1 regulates *notch* receptor genes expression during heart regeneration. By performing RNA in situ analysis on frozen heart sections using either *notch1a*, *notch1b*, *notch2*, or *notch3* probes, we found that inhibition of Brg1 in endothelial cells (DN) resulted in slight upregulation in sham-operated hearts, but had strong induction of *notch1a*, *notch1b*, *notch2*, and *notch3* in injured hearts at 7 dpa compared with control sibling hearts (Ctrl) (Fig. 3a). Furthermore, RNAscope in situ hybridization showed that *notch1b* overlapped with *kdrl*-positive endothelial cells but rarely with *tcf21*-positive epicardial cells in Ctrl hearts (Supplementary Fig. 6a, c) and DN hearts (Supplementary Fig. 6b, d). Also, *notch2* was induced in both *kdrl*- and *tcf21*-positive cells of DN hearts compared with Ctrl hearts (Supplementary Fig. 6e–h). In addition, qRT-PCR of FACS-sorted *kdrl*:*EGFP*-positive endothelial cells from CtrlK and DNK hearts at 7 dpa showed that, compared with CtrlK group, the expression levels of *notch1a*, *notch1b*, *notch2*, and *notch3*, as well as Notch ligands *dll4*, significantly increased in DNK group (Fig. 3b) in the presence of 4-HT. Together, these data suggest an inhibitory effect of Brg1 on the expression of *notch* genes during heart regeneration.

We then investigated how Brg1 regulated *notch* receptor genes. Genome-wide H3K4me3 ChIP-seq data showed that the H3K4me3 levels and peaks were increased in the promoters of *notch1a*, *notch1b*, *notch2*, and *notch3* genomic loci in the DN group compared with those in the Ctrl group (Fig. 3c). Particularly, the promoter regions of *notch1a*, *notch1b*, and *notch2* occupied with the Overlapped Peaks and the peaks signals were stronger in the DN group compared with Ctrl group; and the promoter region of *notch3* had an H3K4me3 peak in DN group that was not in Ctrl group (Fig. 3c). We then used ChIP-qPCR to further confirm the levels of H3K4me3 modification in each of the *notch* promoter regions. ChIP with H3K4me3 antibody and quantitative PCR (ChIP-qPCR) showed that the levels of H3K4me3 of all four *notch* promoter regions were higher in DN hearts than in Ctrl hearts at 7 dpa in the presence of 5 μM 4-HT for 3 days before surgery (Fig. 3d), which was consistent with the elevated expression levels of these genes upon endothelial Brg1 inhibition (Fig. 3a, b). Furthermore, ChIP-qPCR with Brg1 antibody showed that Brg1 bound to the promoter regions of *notch1a*, *notch1b*, *notch2*, and *notch3* (Fig. 3e), suggesting that Brg1 is involved in regulating the H3K4me3 modifications in the *notch* promoters.

### Abnormally activated Notch signaling is responsible for the reduced cardiomyocyte proliferation in DN-xBrg1 hearts

**Fig. 4:**
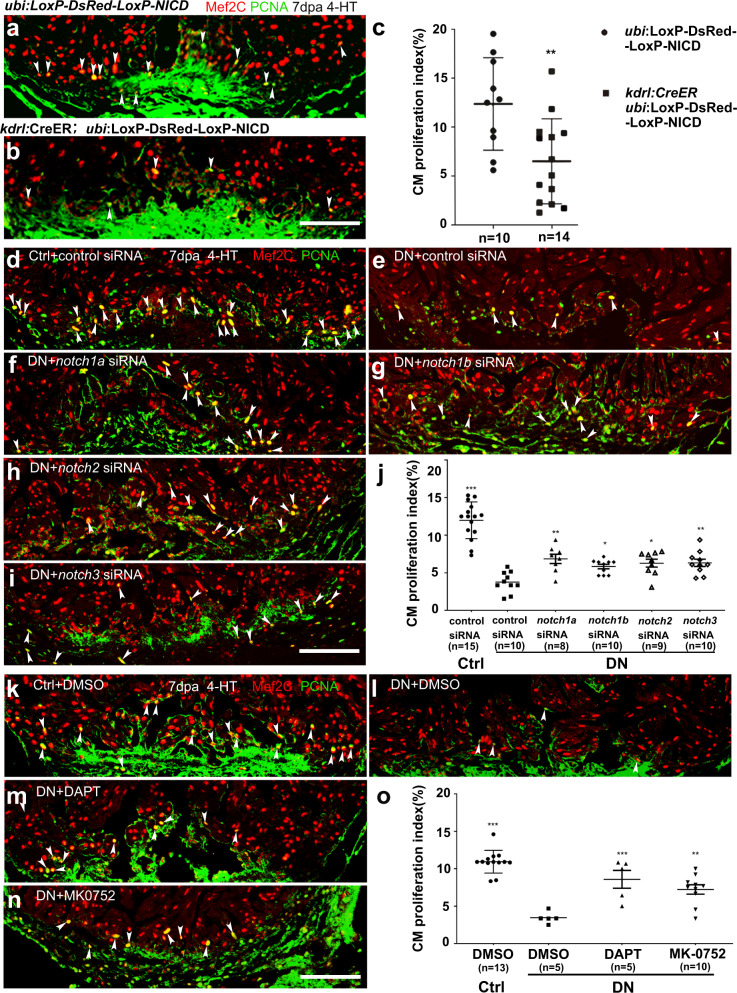
Endothelium-specific expression of *NICD* or *dn-xbrg1* decreases cardiomyocyte proliferation that is partially rescued by inhibition of Notch signaling. **a**, **b** Immunostaining showing that Mef2C^+^ and PCNA^+^ proliferating cardiomyocytes of control (**a**) and endothelial *NICD*-overexpressing heart sections (**b**) at 7 dpa after 4-HT induction. **c** Statistics of panels (**a**) and (**b**) (data are the mean fold change ± s.e.m.; **p < 0.01, unpaired *t*-test). **d**–**i** Representative images of immunostaining showing that, compared with control siRNA treatment (**d**), PCNA^+^/Mef2C^+^ proliferating cardiomyocytes decreased at 7 dpa in DN-xBrg1 hearts (DN) treated with control siRNA (**e**), which were partially rescued by either *notch1a* (**f**), *notch1b* (**g**), *notch2* (**h**), or *notch3* (**i**) siRNA treatment in the presence of 4-HT. Scale bar, 100 μm. **j** Statistics of panels (**d**–**i**) (data are the mean ± s.e.m.; *p < 0.05; **p < 0.01; ***p < 0.005; one-way analysis of variance [*F* = 32.5] followed by Dunnett’s multiple comparison test). **k**–**n** Representative images of immunostaining at 7 dpa showing that, compared with DMSO treatment (**k**), PCNA^+^/Mef2C^+^ proliferating cardiomyocytes in DN mutant hearts decreased (**l**), which were partially rescued by either DAPT (**m**) or MK-0752 treatment (**n**) in the presence of 4-HT. Scale bar, 100 μm. **o** Statistics of panels (**k**–**n**) (data were the mean ± s.e.m.; ***p < 0.005; one-way analysis of variance [*F* = 22.4] followed by Dunnett’s multiple comparison test). *n* number shown here (**c**, **j**, **o**) indicated biological replicates.

Since a previous study has shown that hyperactivation of Notch signaling impairs cardiomyocyte proliferation and heart regeneration^11^, we suspected that abnormally activated Notch signaling might contribute to defects of cardiomyocyte proliferation and regeneration in the endothelium-specific DN-xBrg1 hearts. We generated *Tg(ubi:LoxP-DsRed-STOP-LoxP-NICD; kdrl:CreER)* transgenic fish line to carry out tamoxifen-inducible overexpression of NICD (zebrafish *notch1b* intracellular domain) that specifically activated Notch signaling in endothelial cells. Compared with control hearts at 7 dpa, we found that hyperactivation of Notch signaling in endothelial cells decreased the numbers of PCNA^+^/Mef2C^+^ proliferating cardiomyocytes (Fig. 4a–c), which was consistent with the previous report^11^. We then asked whether simultaneous knockdown of *notch* receptors could rescue the numbers of proliferating cardiomyocytes in DN-xBrg1 mutant hearts. To validate the efficacy of nanoparticle-encapsulated siRNAs mediated gene knockdown, we first confirmed that siRNA could be delivered into endothelial/endocardial cells, epicardial cells, and cardiomyocytes (Supplementary Figs. 7 and 8). We then injected either *notch1a*, *notch1b*, *notch2*, *notch3*, or control siRNA at 1 dpa, analyzed expression levels of the target genes at 2 dpa by performing qRT-PCR, PCR, and RNAscope or RNA in situ hybridization (Supplementary Figs. 9a, c, 10, and 11). All the data showed that the siRNAs used here could decrease expression levels of the corresponding genes. As described above, control and DN zebrafish were infused with 5 μM 4-hydroxytamoxifen (4-HT) for 3 days before surgery, and nanoparticle-encapsulated *notch1a*, *notch1b*, *notch2*, *notch3*, or control siRNA was, respectively, injected every day after surgery until the hearts were harvested at 7 dpa. With control siRNA injection, we found that the PCNA^+^/Mef2C^+^ proliferating cardiomyocytes were fewer in DN hearts than in Ctrl hearts (Fig. 4d, e, j). Interestingly, either *notch1a*, *notch1b*, *notch2*, or *notch3* siRNA was able to partially rescue the numbers of PCNA^+^/Mef2C^+^ proliferating cardiomyocytes in DN zebrafish hearts at 7 dpa, but was unable to return them to the control level (Fig. 4d–j), suggesting that hyperactivation of Notch signaling contributes to defects of myocardial proliferation in DN mutant hearts. In addition, we also chose two chemical inhibitors, DAPT and MK-0752, to interfere with Notch signaling. Compared with DN hearts injected with control DMSO (Fig. 4l), we found more PCNA^+^/Mef2C^+^ proliferating cardiomyocytes in the DN hearts injected with either of the Notch inhibitors (Fig. 4m, n), which was similar to the effect of delivering siRNAs targeting all four *notches* (Supplementary Fig. 12), but fewer than those in Ctrl hearts injected with DMSO (Fig. 4k, o). Thus, these results suggest that abnormally activated Notch signaling is partially responsible for the cardiomyocyte-proliferation defects in the DN mutant hearts. Together, our data suggest that injury-induced endothelial Brg1 negatively regulates the level of H3K4me3 in the promoter regions of *notch1b*, *notch2*, and *notch3* and thus prevents the overactivation of Notch signaling during heart regeneration. When this suppression is released, such as in DN hearts, the level of H3K4me3 modifications in the *notch* promoter regions is abnormally upregulated, resulting in the overactivation of Notch signaling and thus inhibiting regeneration.

### Brg1 interacts with Kdm7aa to fine-tune Notch signaling

**Fig. 5:**
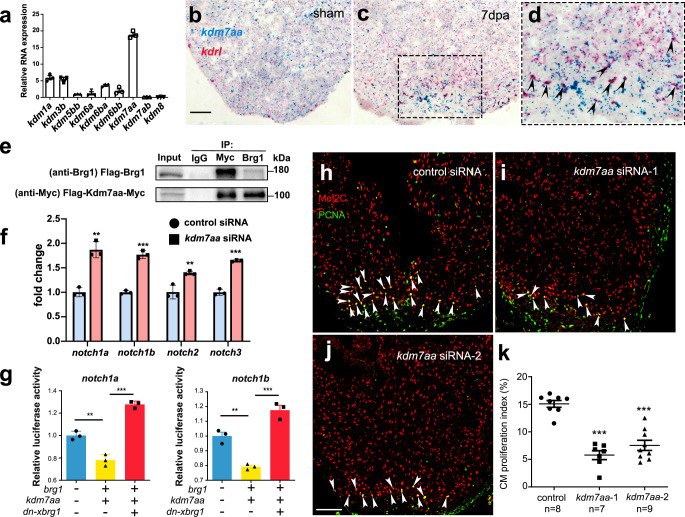
Endothelial Brg1 regulates *notch* receptor expression and myocardial proliferation via interaction with Kdm7aa. **a** Quantitative RT-PCR of *kdm* genes expression, normalized by *gapdh*. **b**–**d** Representative images of RNAscope in situ hybridization with *kdrl* and *kdm7aa* probes (scale bar, 100 μm) and high-magnification image of boxed region in (**c**) (arrowheads, double *kdrl*- and *kdm7aa*-positive endothelial cells). **e** Immunoprecipitation (IP) assays with either anti-Myc or anti-Brg1 antibody in 293T cells. Inputs were used as loading controls and IgG as negative controls. The blots were derived from the same experiment, and they were processed in parallel. **f** Quantitative RT-PCR analysis showing that the expression levels of *notch1a*, *notch1b*, *notch2*, and *notch3* from hearts at 7 dpa injected with encapsulated *kdm7aa* siRNA was higher compared with the control siRNA group. Data represented one of three independent experiments. Data were mean fold changes ± s.e.m., ***p < 0.005, unpaired *t*-test. **g** Luciferase reporter assays in 293T cells stably expressing the *notch* promoter-luciferase reporter in the pGL4.26 vector. Expression plasmid clones containing *kdm7aa*, *brg1*, or *dn-xbrg1* were co-transfected into cells stably expressing each *notch* reporter. Data represented one of two independent experiments, ***p < 0.005, one-way analysis of variance (*F* = 110.6 for the left panel and 60.3 for the right panel) followed by the Bonferroni test. *n* number shown here (**f** and **g**) indicated technical replicates. **h**–**j** Representative images of immunostaining showing the numbers of Mef2C^+^/PCNA^+^ proliferating cardiomyocytes (arrowheads, Mef2C^+^/PCNA^+^ proliferating cardiomyocytes; scale bar, 100 μm). **k** Statistics of panels (**h**–**k**) (*n* numbers indicated biological replicates; data were the mean ± s.e.m.; ***p < 0.005; one-way analysis of variance [*F* = 36.79] followed by Dunnett’s multiple comparison test).

We then asked how Brg1 negatively regulated H3K4me3 modifications in the promoter regions and had its function in regulating *notch* receptor gene expression. It has been reported that Brg1 and histone demethylase (lysine demethylases, KDMs) jointly regulated gene expression in other organs^26–28^. To determine whether KDMs were involved in the regulation of the levels of H3K4me3 by Brg1, we first examined the expression pattern of KDMs during zebrafish heart regeneration. RT-PCR data revealed that *kdm7aa* had the strongest expression while *kdm1a*, *kdm3b*, *kdm5bb*, *kdm6a*, *kdm6ba*, and *kdm6bb*, but not *kdm7ab* and *kdm8*, were weakly expressed in injured hearts at 2 dpa (Fig. 5a). Kdm7aa has been shown to be responsible for histone demethylation at multiple sites, including H3K9, H3K27, H3K36, and H3K20^29^. Interestingly, we also found that *kdm7aa* was induced and enriched in cardiac endothelial cells upon injury using RNAscope with *kdrl* and *kdm7aa* probes (Fig. 5b–d). Therefore, we further examined the interaction between Brg1 and Kdm7aa using immunoprecipitation (IP) in 293T cells. Lysates of cells overexpressing both *Flag-kdm7aa-Myc* and *Flag-brg1* were precipitated by either Myc or Brg1 antibodies. Western blots revealed that IP with either Myc antibody (Myc-tagged Kdm7aa) or Brg1 antibody was able to pull down both Flag-tagged Brg1 (~180 kD) and Myc-tagged Kdm7aa (~100 kD), suggesting that Brg1 physically interacted with Kdm7aa (Fig. 5e, Supplementary Fig. 13a). To examine whether Kdm7aa is involved in Brg1-regulated *notch* receptor gene expression, we utilized nanoparticle-mediated gene-silencing^30,31^ to knock down *kdm7aa* (Supplementary Figs. 7j, k, 8e, 9b, c, 10, and 11c, h). RT-PCR, RNAscope, and RNA in situ hybridization results showed that the downregulation of *kdm7aa* significantly upregulated the expression levels of *notch1a*, *notch1b*, *notch2*, and *notch3* (Fig. 5f, Supplementary Fig. 13b–e). The IP assays performed in the 293T cells indicated the interaction between Kdm7aa and H3K4me3 (Supplementary Figs. 13f and 14a). Western blot data of H3K4me3 in zebrafish hearts revealed that H3K4me3 protein level increased after *kdm7aa* siRNA knockdown compared with control siRNA (Supplementary Figs. 13g and 14b). Consistently, overexpressed zebrafish *kdm7aa* in 293T cells decreased H3K4me3 protein level (Supplementary Figs. 13h and 14c). ChIP Q-PCR analysis showed that H3K4me3 modifications at all four *notches* promoter regions of hearts increased after *kdm7aa* siRNA knockdown compared with control siRNA (Supplementary Fig. 13i). We also used the luciferase reporter system that was driven by the *notch1a* or *notch1b* promoters, and made stable 293T cell lines expressing each of the luciferase reporters. Luciferase assays showed that overexpression of zebrafish *brg1* and *kdm7aa* decreased *notch1a* and *notch1b* reporter activity, while overexpression of *dn-xbrg1* increased the activity of *notch1a* and *notch1b* reporters (Fig. 5g), suggesting a synergistic role of Brg1 and Kdm7aa in controlling the expression levels of *notch* reporter genes. We finally set out to address whether *kdm7aa* was directly involved in regulating zebrafish heart regeneration. We found that the knockdown of *kdm7aa* with two independent siRNAs decreased the numbers of PCNA^+^/Mef2C^+^ proliferating cardiomyocytes compared with control siRNA (Fig. 5h–k). Together, our data suggest that endothelial cell Brg1 interacts with Kdm7aa to maintain the normal activity of *notch* gene promoters, and Kdm7aa modulates the level of H3K4me3 to fine-tune *notch* gene expression during heart regeneration.

## Discussion

In this study, we showed that endothelial Brg1 was required for myocardial proliferation and regeneration in zebrafish; Brg1 interacted with Kdm7aa to fine-tune the level of H3K4me3 in the *notch* receptor promoters and negatively regulated *notch* genes expression during heart regeneration; and Kdm7aa was induced in cardiac endothelial cells and was required for myocardial proliferation. Therefore, our data reveal the role of the endothelial Brg1-Kdm7aa axis in regulating *notch* gene transcription and the essential role of histone methylation via Kdm7aa in myocardial proliferation and regeneration in zebrafish.

Previous studies have shown that Brg1 plays an important role in oocyte genome activation, erythropoiesis, T-cell generation, erythropoiesis, vascular development, nerve development, heart development, and regeneration^21,32–39^. We here demonstrated that conditional inhibition of Brg1 function in endothelial cells, including the endocardium, led to increased cardiac fibrosis and compromised myocardial proliferation and regeneration. Either hypo- or hyperactivation of Notch signaling has been reported to impair cardiomyocyte proliferation and heart regeneration^10–12,40^, suggesting that the precise modulation of Notch family expression is essential for cardiac regeneration. Here, we present several layers of evidence to demonstrate that injury-induced Brg1 and Kdm7aa regulate *notch* gene expression in cardiac endothelium and endocardium. Brg1 and Kdm7aa normally fine-tune the level of the histone marker H3K4me3 in the *notch* gene promoters, thus preventing the abnormal hyperactivation of *notch* receptors after injury. When Brg1 was inhibited in cardiac endothelial cells, the H3K4me3 level increased in the *notch* promoter regions, and *notch* genes were abnormally overexpressed, leading to enhanced cardiac fibrosis and compromised myocardial proliferation and regeneration. Injury-induced expression of *brg1* and *kdm7aa* was evident in cardiac endothelial cells that were consistent with their function, which was further supported by our data on the physical interaction between Brg1 and Kdm7aa, and their function in regulating *notch* promoter activities. Furthermore, either encapsulated siRNA knockdown of *notch* receptors, or chemical Notch inhibitors, partially rescued the phenotype of myocardial proliferation in DN-xBrg1 hearts, further suggesting an important role of Brg1 in regulating *notch* gene expression during heart regeneration. At the same time, how hyperactivated Notch signaling in cardiac endothelium and endocardium represses myocardial proliferation via endocardium–myocardium interaction warrants future investigations.

Chromatin remodeling has been reported to be essential for tissue/organ regeneration in urodeles and zebrafish^17,18^. Brg1 is the major subunit of the SWI/SNF complex and is also an important component of the trithorax group, both of which play essential roles in histone modification, such as the histone markers H3K4me3 (active) and H3K27me3 (repressive). Although data on genome-wide histone acetylation and methylation during organ regeneration are still limited, recent studies suggest that a more open chromatin state is adopted during early fin, retina, and heart regeneration in zebrafish^41–43^. The level of the histone marker H3K4me3 is influenced and catalyzed by lysine methyltransferases of the MLL2 complex and KDMs. Although the MLL2 complex does not provide selective specificity in a particular organ or biological process, it is believed that ATP-dependent chromatin-remodeling proteins such as Brg1 may specifically regulate the “bivalency” state of H3K4me3 and H3K27me3^44^. KDM7 has been reported to act as a dual KDM for histone silencing markers H3K9 and H3K27 in brain development and germ cell genome stability^29,45^, but it is unknown whether it also works for the active histone marker H3K4. We found that *brg1* and *kdm7aa* co-expressed in cardiac endothelial cells upon injury in zebrafish, and they formed a protein complex and functioned synergistically to regulate *notch* receptor gene promoters in mammalian cells. Inhibition of Brg1 function via DN-xBrg1 mutant proteins increased the *notch* promoter activity, suggesting that DN-xBrg1 might replace and/or inhibit Kdm7aa function, and so increased the level of H3K4me3. Our IP data suggest protein interaction between Kdm7aa and H3K4me3, and *kdm7aa* siRNA knockdown or overexpression respectively increased or decreased H3K4me3 modifications. Furthermore, the data on nanoparticle-mediated *kdm7aa* siRNA knockdown supported its function in myocardial proliferation and regeneration. Thus, this work reveals an interesting mechanism on the selective modulation of H3K4me3 by Brg1 and Kdm7aa and their essential function in zebrafish heart regeneration, even though we cannot rule out the possibility that Brg1-Kdm7aa affect the expression of the *notch* receptors in ways beyond H3K4me3 modifications.

## Methods

### Animal models

Male and female zebrafish were raised and handled according to a zebrafish protocol (IMM-XiongJW-3) approved by the Institutional Animal Care and Use Committee at Peking University, which is fully accredited by The Association for Assessment and Accreditation of Laboratory Animal Care International. Wild-type TU, *Tg(kdrl:EGFP)*^46^, *Tg(kdrl:CreER)*^22^, *Tg(fli1:nucEGFP)*^47^, *Tg(tcf21:DsRed)*^21^, *Tg(myl7:cypher-EGFP)*^21^, *Tg(kdrl:mCherry*)^48^, and *Tg(ubi:LoxP-DsRed-STOP-LoxP-dn-xbrg1)* zebrafish^21^ were maintained at 28 °C at a density of four fish per liter. Adult zebrafish were anesthetized in standard E3 medium containing 0.4% tricaine (ethyl 3-aminobenzoate methanesulfonate salt; E10521; Sigma-Aldrich) before ventricular resection as described previously^21^. Animals were randomized into groups for each experiment.

Wild-type C57BL/6 mice were purchased from Vital River Laboratory Animal Technology Co., Ltd (Beijing, China). The mice used in this study were raised and handled according to the animal protocol IMM-XiongJW-4 approved by the Peking University Institutional Animal Care and Use Committee, which is fully accredited by the Association for Assessment and Accreditation of Laboratory Animal Care International. The mice were maintained at the humidity ranges from 40 to 70% and temperature from 21 to 23 °C and with the 12-h light/12-h dark cycle.

### Construction of *Tg(ubi:LoxP-DsRed-LoxP-NICD)* transgenic zebrafish line

To generate the *Tg(ubi:LoxP-DsRed-STOP-LoxP-NICD)* zebrafish line that overexpresses *NICD*, a homologous recombination reaction was conducted with *ubi:LoxP-DsRed-STOP-LoxP-EGFP* plasmid (kindly provided by Dr. C. Geoffrey Burns at Harvard Medical School)^49^ by replacing *EGFP* with zebrafish *notch1b-NICD* cDNA (*N1bICD*). The function of *N1bICD* has been validated as described previously^48^. This *Tol2-NICD* plasmid was made and injected into one-cell stage wild-type embryos together with Tol2 transposase mRNA as described previously^50^. Heterozygous transgenic zebrafish were raised and genotyped for all experiments.

### 4-hydroxytamoxifen (4-HT) treatment

We generated *Tg(ubi:LoxP-DsRed-STOP-LoxP-dn-xbrg1; kdrl:CreER)* mutant (DN) and *Tg(ubi:LoxP-DsRed-STOP-LoxP-dn-xbrg1)* control sibling (Ctrl) adult zebrafish by crossing *Tg(ubi:LoxP-DsRed-STOP-LoxP-dn-xbrg1)* with *Tg(kdrl:CreER)* zebrafish. To induce Cre recombination, adult DN mutant and Ctrl sibling zebrafish were bathed for 24 h in the presence of 5 μM 4-HT (H7904; Sigma) made from a 10 mM stock solution dissolved in 100% ethanol at room temperature. These zebrafish were treated with 4-HT at a density of 3–4 zebrafish per 150 ml system water. Ventricular resection was performed 3 days after 4-HT treatment. Transgenic zebrafish were confirmed by PCR-based genotyping and were randomly selected for all experiments.

### Ventricular resection in adult zebrafish

The ventricular resection was performed according to a well-established procedure^31,51,52^. Briefly, zebrafish were anesthetized with 0.4% tricaine and placed in the groove of a sponge. The pericardial sac was exposed by removing surface scales and a small piece of skin, and the ventricle apex was gently pulled up and removed with Vannas scissors. The zebrafish was quickly placed back into a system water tank, and water was puffed over the gills with a plastic pipette until it breathed and swam regularly. The surface opening sealed automatically within a few days.

### Fluorescence-activated cell sorting (FACS) of cardiac endothelial cells and flow cytometry

Cardiac endothelial cells from *Tg(ubi:LoxP-DsRed-STOP-LoxP-dn-xbrg1; kdrl:EGFP)* control (CtrlK) and *Tg(ubi:LoxP-DsRed-STOP-LoxP-dn-xbrg1; kdrl:CreER; kdrl:EGFP)* mutant (DNK) ventricles at 7 dpa with 4-HT treatment were isolated according to an established protocol^53^. Briefly, ~15 adult zebrafish hearts were isolated and washed in cold PBS with 10 U/ml heparin (H8060; Solarbio). After the atrium and bulbus were removed, the ventricles were carefully cut into small pieces using forceps and collected into 1.5-ml centrifuge tubes containing cold PBS with 5 mM glucose. The sliced tissue was then transferred to a glass tube along with a magnetic stir bar and 1.5 ml digestion buffer in Dulbecco’s modified Eagle’s medium containing collagenase type II (250 U/ml) (17101015; Gibco), collagenase type IV (300 U/ml) (17104019; Gibco), and DNase I (30 μg/ml) (A3778; AppliChem). The tube was then transferred to a 32 °C water bath with stirring and incubated for 1 min. After incubation, the tube was removed from the water bath and left at room temperature until the tissue settled on the bottom. The supernatant was discarded to remove blood cells, followed by washing once with cold PBS. This was followed by a series of digestion steps with 1.5 ml digestion buffer. Each step consisted of 10 min of digestion followed by 3 min of sedimentation. The supernatants were collected in a 15-ml falcon tube containing 2 ml ice-cold PBS. The cell suspensions were centrifuged at 300 g for 5 min at 4 °C, and the cell pellets were gently resuspended in 1 ml PBS. The resuspended cells were strained using a 70-µm cell strainer and kept on ice for FACS. Cardiac endothelial cells were sorted through the GFP channel and were collected into a tube containing 0.1 ml Lysis Buffer from Magen RNA Nano Kit (R4125; Magen) for RNA isolation. Cell sorting was carried out on the Beckman Coulter MoFlo XDP, and flow cytometry was carried out on the Beckman Cytoflex EX S. Data analysis was performed by FlowJo.

### RNA-seq of cardiac endothelial cells

The RNA of heart endothelial cells from CtrlK sibling and DNK mutant ventricles at 7 dpa was purified using a Magen RNA Nano Kit (R4125; Magen). About 30 ng of total RNA was used for next-generation library preparation under the guidelines of the NEBNext Ultra DNA Library Prep Kit for Illumina (E7370; NEB). The libraries were loaded for 2 × 150 bp pair-end sequencing using Illumina Hiseq 2500. Raw reads were preprocessed and quality controlled with FastQC (The Bioinformatics Group, Babraham Institute; Version: 0.11.9). Reads for each library were mapped using HISAT2 (Version: 2.2.1)^54^ against the zebrafish reference genome assembly GRCz11 with default parameters. Uniquely mapped reads were extracted to calculate the read counts of each gene, using the matching gene annotation (http://ftp.ensembl.org/pub/release-103/gtf/danio_rerio/Danio_rerio.GRCz11.103.gtf.gz; v. 103) from Ensembl with FeatureCounts (Version: 2.0.1)^55^. Genes were further filtered, and those with low expression in all samples (FPKM < 0.5 in all samples) were removed from differential gene expression analysis. Differential analysis was conducted with DEseq2^56^. Genes with an adjusted *p*-value <0.05 were taken as significantly differentially expressed genes in the DNK condition compared with CtrlK. FPKM values were calculated with Stringtie (Version: 2.1.5)^57^, and normalized *Z*-score values were used to draw heatmaps using the ComplexHeatmap R package^58^. Gene ontology enrichment analysis for the differentially expressed genes was performed with clusterProfiler tool (R package, version: 3.18.1)^59,60^. All expressed genes in either CtrlK or DNK condition were used as the background. GO terms with BH adjusted *p*-value <0.01 were taken as enriched GO terms. The top ten enriched GO terms were shown as barplot for biological process, cellular component, and molecular function. Sequencing data have been deposited in GEO under accession code GSE200936.

### ChIP-seq

ChIP-seq libraries were prepared using the VAHTS Universal Pro DNA Library Prep Kit (ND608; Vazyme) for Illumina. Five nanograms of DNA was used as starting material for input and IP samples. Libraries were amplified using 13 cycles on the thermocycler. Post-amplification libraries were size selected at 250–450 bp in length using Agencourt AMPure XP beads (A63880; Beckman Coulter). Libraries were validated using the High Sensitivity DNA Kit (5067-4626; Agilent) and loaded for pair-end sequencing using Illumina NovaSeq 6000. Trimmomatic tool (https://github.com/usadellab/Trimmomatic; Version: 0.39) was used to trim reads with a quality drop below a mean of Q15 in a window of 5 nucleotides and reads with length below 15 nucleotides were filtered out. After the quality control step, the trimmed and filtered reads were aligned to the Zebrafish reference genome GRCz11 using STAR (https://github.com/alexdobin/STAR; Version: 2.7.8a) with the parameters “--outFilterMismatchNoverLmax 0.2-outFilterMatchNmin 20 --alignIntronMax 1 --outFilterMultimapNmax 1” to retain only unique alignments. Reads were deduplicated using Picard (Broad Institute; Version: 2.25.0) to remove PCR artifacts. Since the numbers of H3K4me3 peaks may be affected by the sequencing depths, we used the same number of reads (17.5 million pairs) randomly selected from samples of each condition for downstream analysis. The MACS2 peak caller (https://github.com/macs3-project/MACS; Version: 2.2.7.1) was employed for each condition with parameters “-q 0.0001 -broad -nomodel -nolambda”. Peaks not located in defined chromosomes were further removed. The filtered peaks were used to do the downstream analysis. Intersection between peaks in CtrlK and DNK conditions was performed with Bedtools toolkit (Version: 2.30.0)^61^. Normalized read coverages and subtraction of read coverage were calculated with deeptools toolkit (Version: 2.5.3)^62^. ChIPseeker^63^ was performed to display the genomic distribution of H3K4me3 peaks based on the matching gene annotation (v. 103) from Ensembl. Peaks for different groups were annotated with ChIPseeker package (version: 1.26.2)^63^. GO enrichment analysis was further performed for genes closely located near peaks of each group with clusterProfiler package (version: 3.18.1). The H3K4me3 ChIP-seq traces were represented in IGV (Integrative Genomics Viewer) browser^64^. Sequencing data have been deposited in GEO under accession code GSE200937.

### Quantitative RT-PCR analysis

For FACS-sorted cardiac endothelial cells, RNA from CtrlK sibling and DNK mutant ventricles at 7 dpa was purified using a Magen RNA Nano Kit (R4125; Magen). About 20 ng RNA was used for reverse transcription with MALBAC RNA amplification Kit (KT110700424; YIKON GENOMICS)^65^. For RNA extraction from whole hearts, an RNeasy Mini Kit (74106; Qiagen) was used to purify RNA, and 500 ng RNA was used for reverse transcription with a Prime Script RT Reagent Kit (RR037A; Takara). Quantitative PCR was performed using TB Green Premix DimerEraser Kit (RR091A; Takara) or ChamQ Universal SYBR qPCR Master Mix (Q711; Vazyme). The primer sequences are listed in Supplementary Table 1.

### Delivery of chemical Notch inhibitors and siRNAs into adult zebrafish heart

siRNAs were encapsulated in nanoparticles and then injected into the pericardial sac as described previously^30,31,66,67^. To evaluate the effect of siRNA-mediated rescue on cardiomyocyte proliferation, 10 μl polyethylene glycol-polylactic acid nanoparticle-encapsulated siRNAs was injected into the pericardial sac daily from 2 to 7 dpa. The Notch inhibitors MK-0752 (HY-10974; MCE) and DAPT (A07D5942; Sigma) were first dissolved in DMSO to make a 20 mM stock solution. Before injection, the stock was diluted to the working concentration (30 μM), and 10 μl of diluted inhibitor was injected daily from 4 to 6 dpa. The injected hearts at 7 dpa were then collected for subsequent experiments. siRNA sequences for *notch1a*, *notch1b*, *notch2*, *notch3*, and *kdm7aa* are listed in Supplementary Table 2.

### RNAscope and RNA in situ hybridization, immunostaining, and histology

RNAscope (Advanced Cell Diagnostics, Hayward, CA) was applied to 10-μm sections from freshly frozen hearts embedded in Optimal Cutting Temperature (OCT) compound (4583; Sakura). Fresh tissue was fixed in 10% prechilled neutral buffered formalin in 1 × PBS at 4 °C, followed by dehydration, and then treated with RNAscope® hydrogen peroxide (in RNAscope Universal Pretreatment Kit; 322380; ACD) for 10 min at room temperature. The slides were washed with water and incubated with RNAscope Protease IV (in RNAscope Universal Pretreatment Kit; 322380; ACD) for 30 min at room temperature. Then, they were washed five times in 1 × PBS, and the RNAscope® 2.5 HD Duplex Detection Kit (322430; ACD) was applied to visualize hybridization signals. The RNAscope Probes used in this work included RNAscope Probe-Dr-*smarca4a* (457431; ACD), RNAscope Probe-Dr-*kdm7aa* (822391; ACD), RNAscope Probe-Dr-*notch1b* (431941; ACD), RNAscope Probe-Dr-*notch2* (590431, ACD), RNAscope Probe-Dr-*kdrl*-C2 (416611-C2; ACD), RNAscope Probe-Dr-*tcf21*-C2 (485341-C2; ACD), and RNAscope Probe-Dr-*coro1a*-C2 (496571-C2; ACD). Three injured and sham-operated hearts were used for each RNAscope in situ hybridization.

RNA in situ hybridization was performed on 10-μm sections from fixed frozen hearts embedded in OCT compound. To generate RNA probes, we amplified *notch1a*, *notch1b*, *notch2*, and *notch3* cDNA from regenerating hearts at 7 dpa, blunt-ligated cDNA into a pEASy-Blunt vector, and generated digoxigenin-labeled RNA probes using T7 RNA polymerases (M0251; NEB). In situ hybridization was performed on cryosections of 4% paraformaldehyde-fixed hearts as previously described^68^.

For immunofluorescence staining, adult zebrafish hearts were fixed in 4% paraformaldehyde at room temperature for 2 h, dehydrated, and embedded in paraffin and sectioned at 5 μm. The sections were dewaxed, rehydrated, and washed in 1 × PBS. The antigens were repaired with the citric acid buffer (CW0128S; CWBIO). After washing, the sections were blocked in 10% FBS in PBST (1% Tween 20 in PBS) and then incubated with diluted primary antibodies (in PBST containing 10% FBS) overnight at 4 °C. The primary antibodies used for immunofluorescence were anti-Mef2c (HPA005533; Sigma; 1:200), anti-GFP (A-11122; Invitrogen; 1:500), anti-PCNA (P8825; Sigma; 1:300), anti-myosin heavy-chain monoclonal antibody (14-6503-82; eBioscience; 1:500), anti-DsRed (BE3307; EASYBIO; 1:300) and the Brg1 antibody (1:250), which was raised against a glutathione S-transferase-BRG1 fusion protein (human BRG1 amino-acids 1086–1307)^69,70^. After washing, the sections were incubated with secondary antibodies for 2 h at room temperature. The secondary antibodies (1:300 diluted in PBST containing 10% FBS) were Alexa Fluor 488 goat anti-mouse IgG (A21121; Invitrogen; 1:300), Alexa Fluor 488 goat anti-rabbit IgG (A11034; Invitrogen; 1:300), Alexa Fluor 555 goat anti-mouse IgG (A21424; Invitrogen; 1:300), and Alexa Fluor 555 goat anti-rabbit IgG (A21428; Invitrogen; 1:300).

RNA and RNAscope in situ hybridization was examined under a DM5000B microscope (Leica, Germany); immunofluorescence images were captured on a confocal microscope (LSM510 or LSM980; Carl Zeiss, Germany). The signal intensity or area was quantified using MBF ImageJ (https://imagej.nih.gov/ij/)^71^.

### Acid fuchsin orange G staining (AFOG)

AFOG staining was performed on paraffin sections following the manufacturer’s instructions^51^. The sections were incubated in Bouin’s solution (HT10132; Sigma) at 56 °C for 2.5 h, and at room temperature for 1 h, washed in tap water, incubated in 1% phosphomolybdic acid (P4869; Sigma) for 5 min, washed with water, and then stained with AFOG solution consisting of 3 g acid fuchsin (F8129; Sigma), 2 g orange G (O3756, Sigma), and 1 g aniline blue (AB0083; BBI) dissolved in 200 ml acidified distilled water (pH 1.1) for 10 min. The sections were rinsed with distilled water, dehydrated, mounted, and staining was photographed under a DM5000B microscope (Leica, Germany).

### Chromatin immunoprecipitation (ChIP) and quantitative ChIP (qChIP)

About 25 zebrafish hearts were pooled for each ChIP experiment. The hearts were dissected from adult zebrafish, and the outflow tract and atrium were removed. Chromatin isolation and ChIP assays were performed using a Pierce Magnetic ChIP Kit (26157; Pierce). Anti-Brg1 (5 µl for each reaction) and anti-H3K4me3 (Ab8580; Abcam; 5 µl for each reaction) antibodies were used for the ChIP assays. The Brg1 antibody used here was a mouse polyclonal antibody raised against glutathione S-transferase (GST)-tagged zebrafish Brg1 amino acid 1098–1286 that was made in our laboratory. The purified antigen protein was provided by HUABIO, HangZhou. Mice were inoculated with purified antigens, and their serum was collected as polyclonal antibodies. The DNA bound by ChIP was used for library construction and quantitative PCR. The primer sequences are listed in Supplementary Table 2.

### Immunoprecipitation (IP)

The full-length coding cDNA of zebrafish *kdm7aa* was isolated from the regenerating heart cDNA library and cloned into the pcDNA3.1 vector. For co-IP, 293T cells (CRL-1573; American Type Culture Collection) were transfected with pcDNA3.1-*Flag-kdm7aa-Myc* and pcDNA3.1*-Flag-brg1* using Fugen HD Transfection Reagents (2311; Promega), and after 48 h the transfected cells were lysed in NP-40 lysis buffer (P0013F; Beyotime). After brief centrifugation, the supernatants were collected for immunoprecipitation, while a protein extraction fraction was set aside for input controls. Equal volumes of supernatants were incubated overnight with either anti-Myc (AT0023; Engibody; 5 µg for each reaction), anti-Flag (F3165; Sigma; 5 µg for each reaction), anti-IgG (PI31160; Thermo Fisher Scientific; 5 µg for each reaction), and anti-Brg1 (10 µl for each reaction). The next morning, 25 μl of Pierce Protein A/G Magnetic Beads (88802; Pierce) were added and incubated with the IP mixture for 2 h at room temperature. The beads were then washed for 5 min and repeated three times in IP wash buffer (30 mM HEPES, 100 mM NaCl, 1 mM EDTA, 0.5% NP-40, pH 7.5), and were subsequently eluted with 1× loading buffer with heating at 100 °C for 10 min. The Brg1 antibody used here was a mouse polyclonal antibody raised against GST-tagged zebrafish Brg1 amino acid 1098–1286, as described above.

### Zebrafish *notch* promoter-luciferase assays

The promoter sequences of *notch* receptors were cloned into the luciferase reporter vector pGL4.26, with the *notch1a* promoter (from 171 bp to +3 bp) and *notch1b* promoter (from −41 bp to +58 bp), of which the ATG was considered to be +1 bp. Stable 293T cell lines (CRL-1573; American Type Culture Collection) for each of the four *notch* reporters were generated in the presence of 150 μg/ml hygromycin B. Isolated reporter cells for each of the *notch* receptors were co-transfected with pcDNA3.1-*brg1*, pcDNA3.1-*kdm7aa*, pcDNA3.1-*dn*-*xbrg1*, and pREP4-*Renilla*. Luciferase assays were carried out at 48 h after infection following the manufacturer’s instructions with the Dual-luciferase Reporter Assay System (E1910; Promega). Firefly luciferase activity was normalized by *Renilla* luciferase activity.

### Statistical analysis

All statistics were calculated using Statistical Product and Service Solutions (SPSS) software from IBM or GraphPad Prism. The statistical significance of differences between the two groups was determined using the independent unpaired *t*-test, with two-tailed *p*-values, and the data are reported as the mean ± s.e.m. Among three or more groups, one-way analysis of variance followed by Bonferroni’s multiple comparison test or Dunnett’s multiple comparison test was used for comparisons.

### Reporting summary

Further information on research design is available in the Nature Research Reporting Summary linked to this article.

## Supplementary information

Supplementary Information

Dataset 1: Supplementary Table 3

Dataset 2: Supplementary Table 4

Reporting Summary

## Data Availability

Sequencing data that support the findings of this study were deposited into the GEO with the accession codes GSE200936 and GSE200937. The non-sequencing data and/or materials generated during this study are available from J.-W.X. upon request.

## Code availability

Only public software and custom code were used to generate or process datasets in this work. The software version, references, and parameters were well described in the “Methods” section.
