# Supplementary material for: Endothelial Brg1 fine-tunes Notch signaling during zebrafish heart regeneration
Source: NPJ Regen Med. 2023 Apr 7;8:21. doi: 10.1038/s41536-023-00293-4 (PMC10082087; doi:10.1038/s41536-023-00293-4)
Supplement: Supplementary file 1 — Supplementary Information [file 41536_2023_293_MOESM1_ESM.pdf]

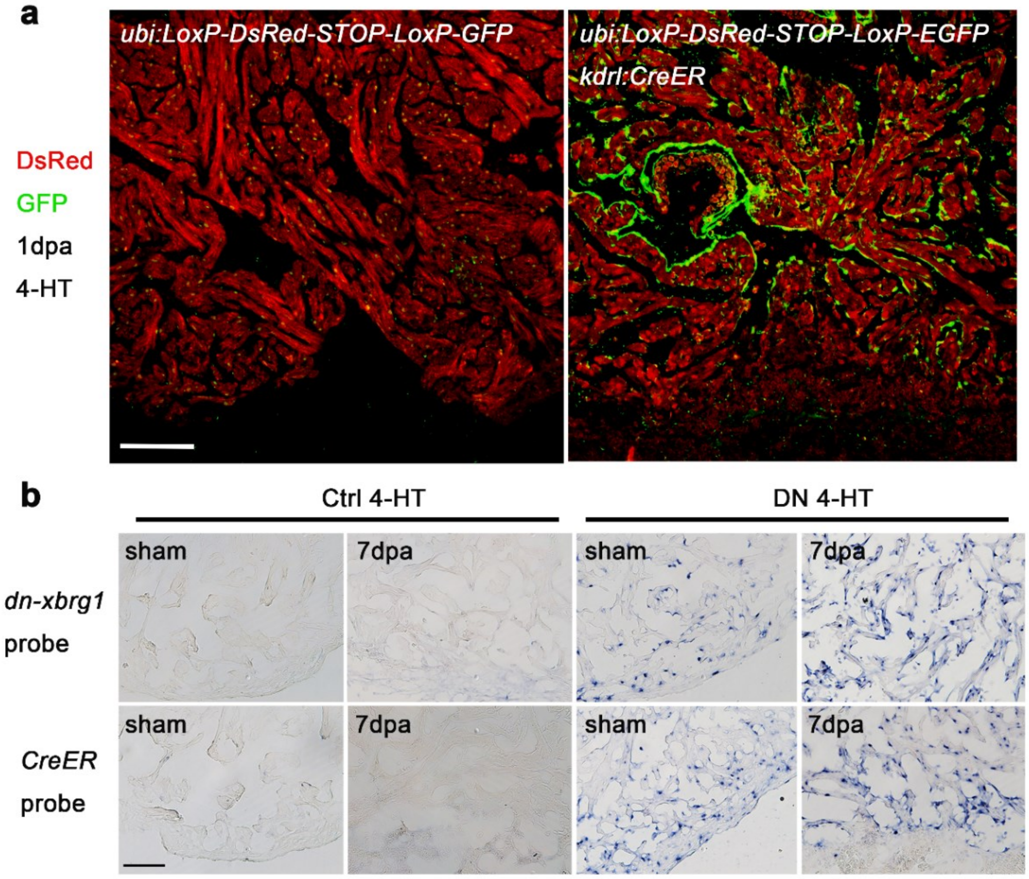

Supplementary Figure 1. Validation of endothelial-specific expression of *kdrl:CreER* after Cre recombination.

a Immunofluorescence with anti-DsRed and anti-GFP of heart sections from control siblings *Tg(ubi:LoxP-DsRed-STOP-LoxP-EGFP)* and endothelium-specific GFP-overexpressing hearts *Tg(ubi:LoxP-DsRed-STOP-LoxP-EGFP; kdrl:CreER)* at 1 dpa after 4-HT induction. Noting the GFP expression after Cre recombination. **b** Representative images of RNA *in situ* hybridization with *dn-xbrg1* and *CreER* probes on frozen sections of sham-operated Ctrl hearts, injured Ctrl hearts, sham-operated DN hearts, and injured DN hearts at 7 dpa. Scale bars, 100 μm.

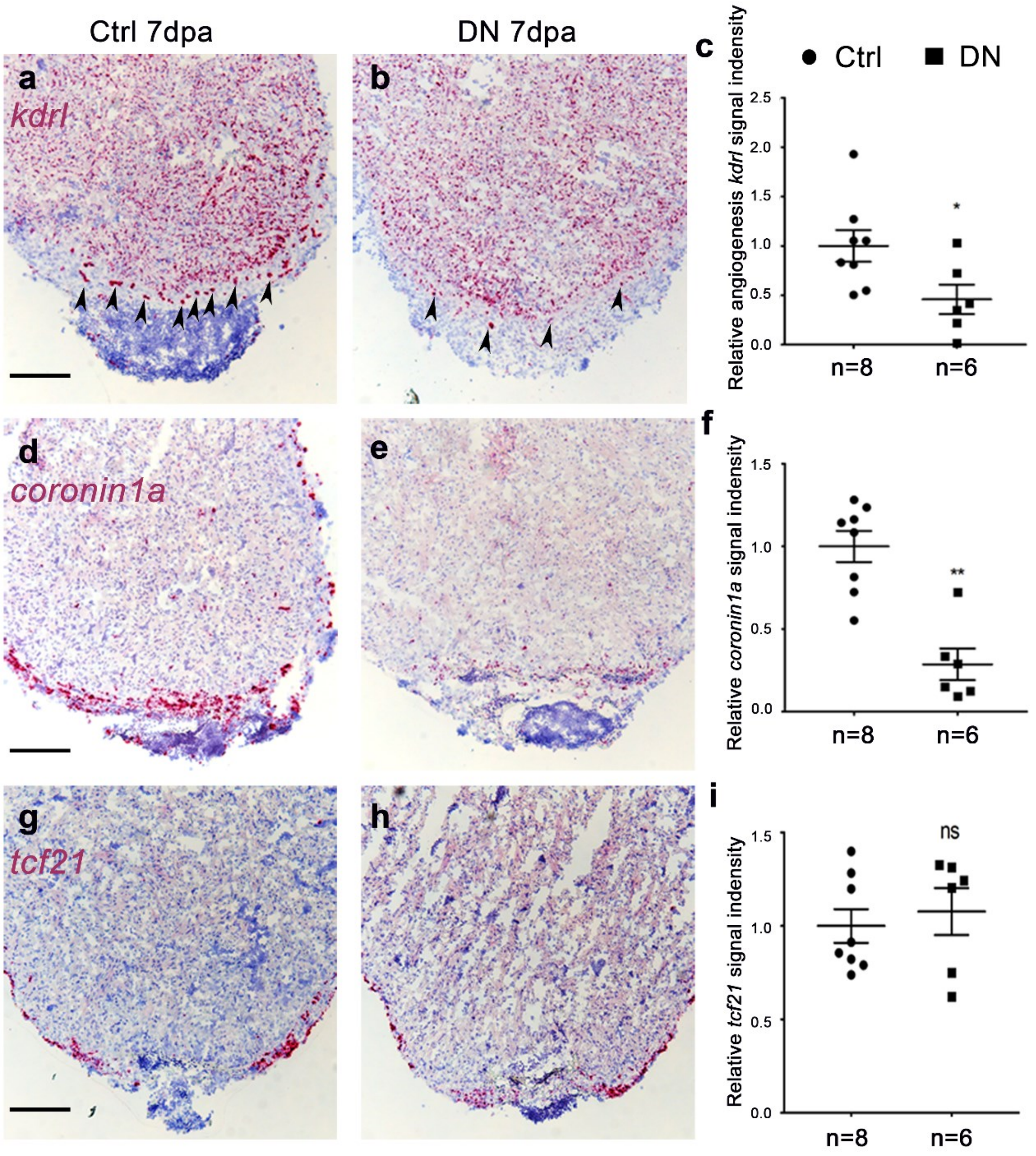

Supplementary Figure 2. Endothelium-specific inhibition of Brg1 impairs angiogenesis and immune responses but not epicardial activation.

a, b, d, e, g, h RNAscope *in situ* hybridization on representative sections of control sibling hearts [Ctrl: *Tg(ubi:LoxP-DsRed-STOP-LoxP-DN-xbrg1)*] (a, d, g) and DN-xBrg1 mutant hearts [DN: *Tg(ubi:LoxP-DsRed-STOP-LoxP-DN-xbrg1; kdrl:CreER)*] (b, e, h) at 7 dpa, using *kdrl* (endothelial cell marker) (a-b), *coronin1a* (leukocyte marker) (d-e), and *tcf21* (epicardium marker) probes (g-h). Note that endothelium-specific inhibition of Brg1 interfered with *kdrl*-positive endothelial cells (arrowheads) and *coronin1a*-positive leukocyte recruitment while having no effects on *tcf21*-positive epicardium in the presence of 4-HT (scale bars, 100 μm). **c, f, i** Statistics of panels a and b (c), d and e (f), and g and h (i). Data were the mean ± s.e.m; *p <0.05, **p <0.01, ns, not significant, unpaired *t*-test. N number shown here (c, f, i) indicated biological repetition.

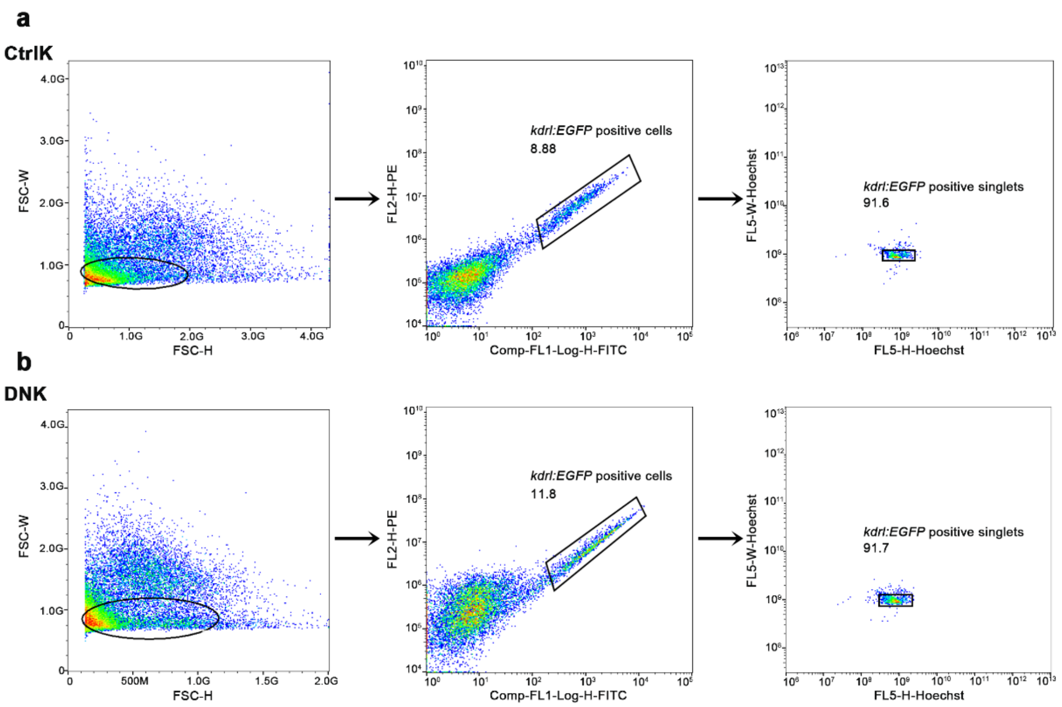

Supplementary Figure 3. *kdrl:EGFP* cells sorting strategy. a, b The gating strategy for acquiring *kdrl:EGFP* positive cells from CtrlK (a) and DNK (b) groups for the subsequent RNA isolation, RNA-seq and qRT-PCR correspond to Figure 2a and 3b.

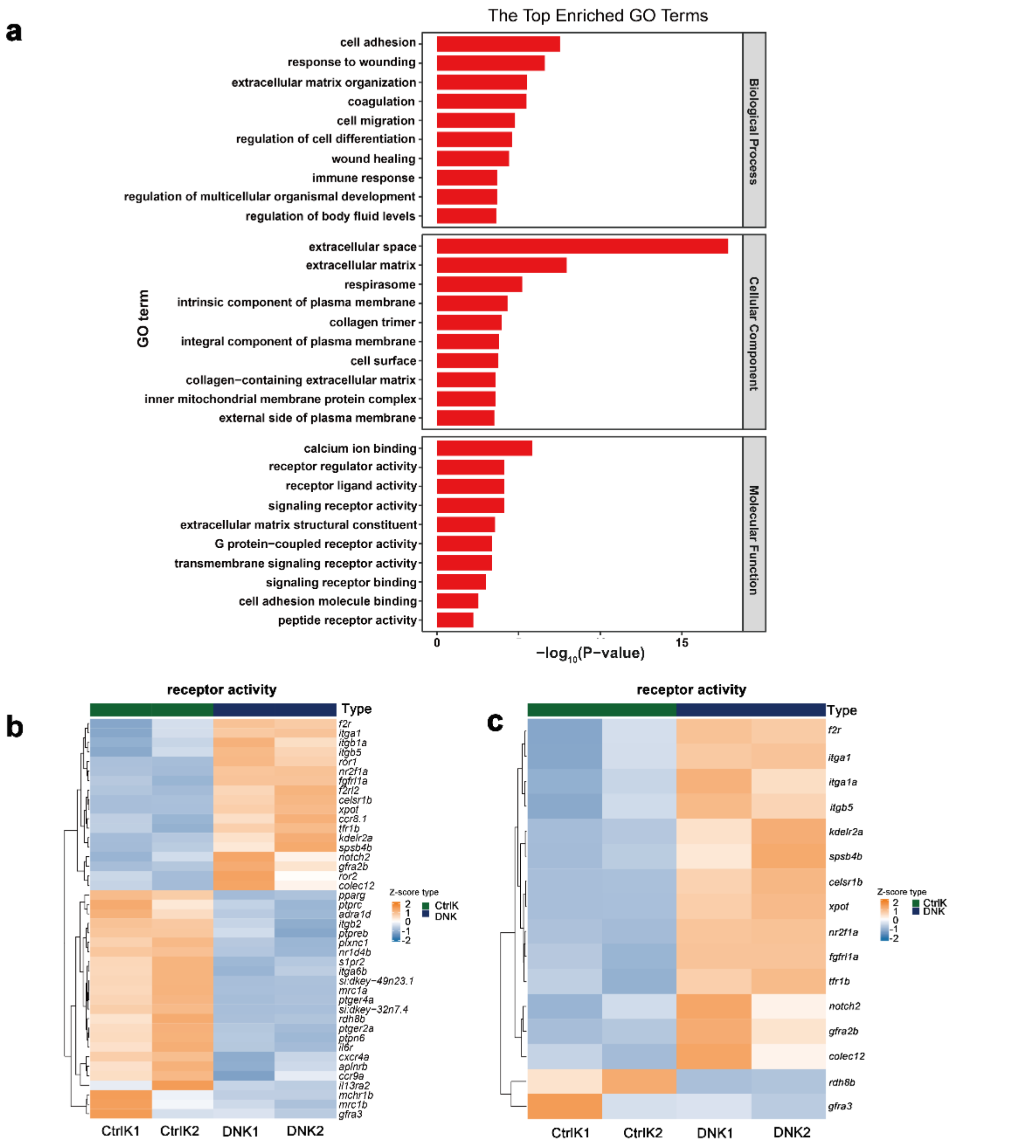

Supplementary Figure 4. RNA-sequencing analysis reveals that endothelial-specific inhibition of Brg1 affects a cluster of receptor genes expression.
a Gene Ontology (GO) enrichment analysis of differentially expressed genes in the DNK group compared with CtrlK group. The top ten enriched GO terms were shown for Biological Process, Cellular Component, and Molecular Function, respectively. **b** Heat map displaying Z-score normalized gene expression for receptor activity related genes (GO:0004872), which were differentially expressed (adjusted P-value < 0.05 from DEseq2 result) in FACS-sorted *kdrl*-eGFP positive endothelial cells between *Tg(ubi:LoxP-DsRed-STOP-LoxP-DN-xbrg1; kdrl:CreER; kdrl:EGFP)* dominant-negative Brg1 hearts (DNK1 and DNK2) and *Tg(ubi:LoxP-DsRed-STOP-LoxP-DN-xbrg1; kdrl:EGFP)* control hearts (CtrlK1 and CtrlK2) at 7 dpa in the presence of 4-HT. Columns represented individual samples (two biological replicates for each condition). **c** Heat map showing expression of receptor activity related genes that were not only differentially expressed in FACS-sorted *kdrl-EGFP* positive cells between DNK and CtrlK hearts, but were also marked by differentially occupied H3K4me3 peaks in their promoters.

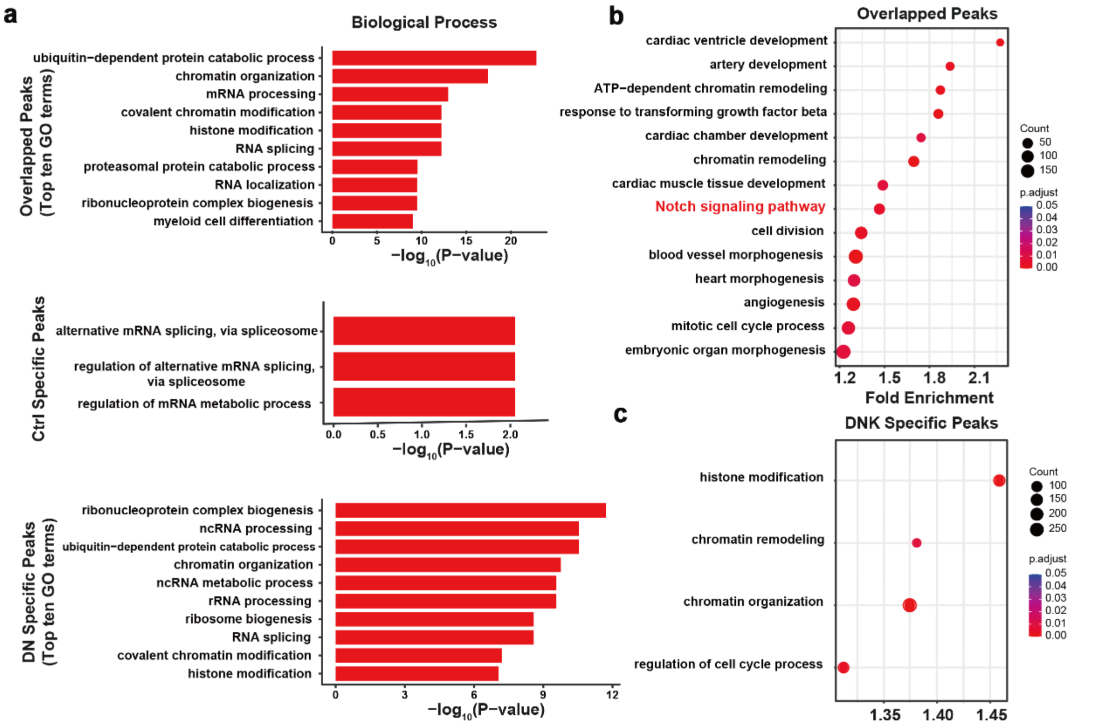

Supplementary Figure 5. GO enrichment analysis for ChIP-seq data

a Gene Ontology (GO) enrichment analysis of genes closely located near **Overlapped Peaks** (up panel), **Ctrl Specific Peaks** (middle panel) and **DN Specific Peaks** (down panel). The top ten enriched GO terms in biological process were shown for overlapped peaks and DN specific peaks.

b Dot plot of GO analysis for genes near overlapped peaks. Shown were the relevant significant enriched categories for Biological process. GO term related to Notch signaling pathway was highlighted in red.

c Dot plot of GO analysis for genes near DN specific peaks. Shown were the relevant significantly enriched categories for Biological process.

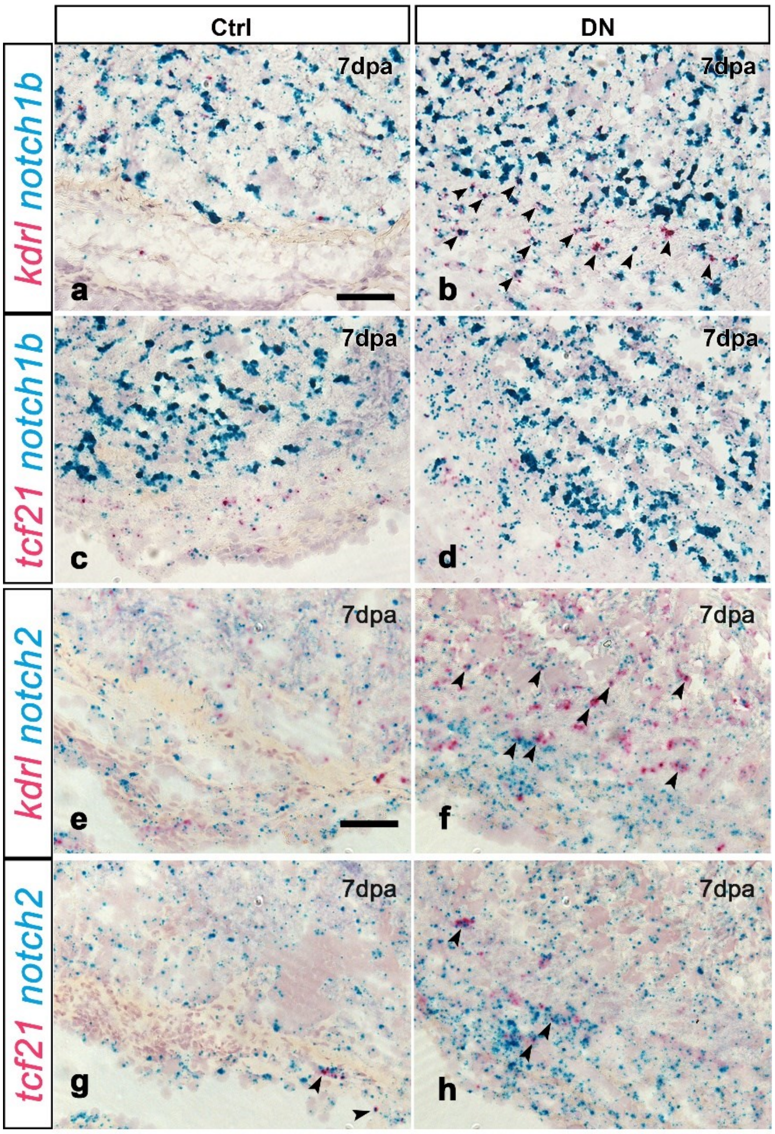

Supplementary Figure 6. *notch1b* and *notch2* are induced in endothelial cells of hearts expressing DN-xBrg1 after ventricular resection.

a-h RNAscope *in situ* hybridization of heart sections from control sibling (Ctrl) (a, c, e, g) and dominant-negative Brg1 (DN) hearts (b, d, f, h) at 7 dpa, using either *kdrl* (endothelial cell marker) (a, b, e, f) or *tcf21* (epicardium marker) (c, d, g, h) probes to co-stain with *notch1b* probes (a-d) or *notch2* probes (e-h). Note that *notch1b* was particularly induced in *kdrl*-positive endothelial cells, but rarely in *tcf21*-positive epicardium of DN hearts compared with Ctrl hearts, and *notch2* was induced in *kdrl*-positive and *tcf21*-positive cells of DN hearts compared with Ctrl hearts in the presence of 4-HT (arrowheads, double-positive signals for *kdrl*- and *notch1b-positive endothelium, kdrl*- and *notch2*-positive endothelium, and *tcf21*- and *notch2*-positive epicardium; scale bars, 50 μm).

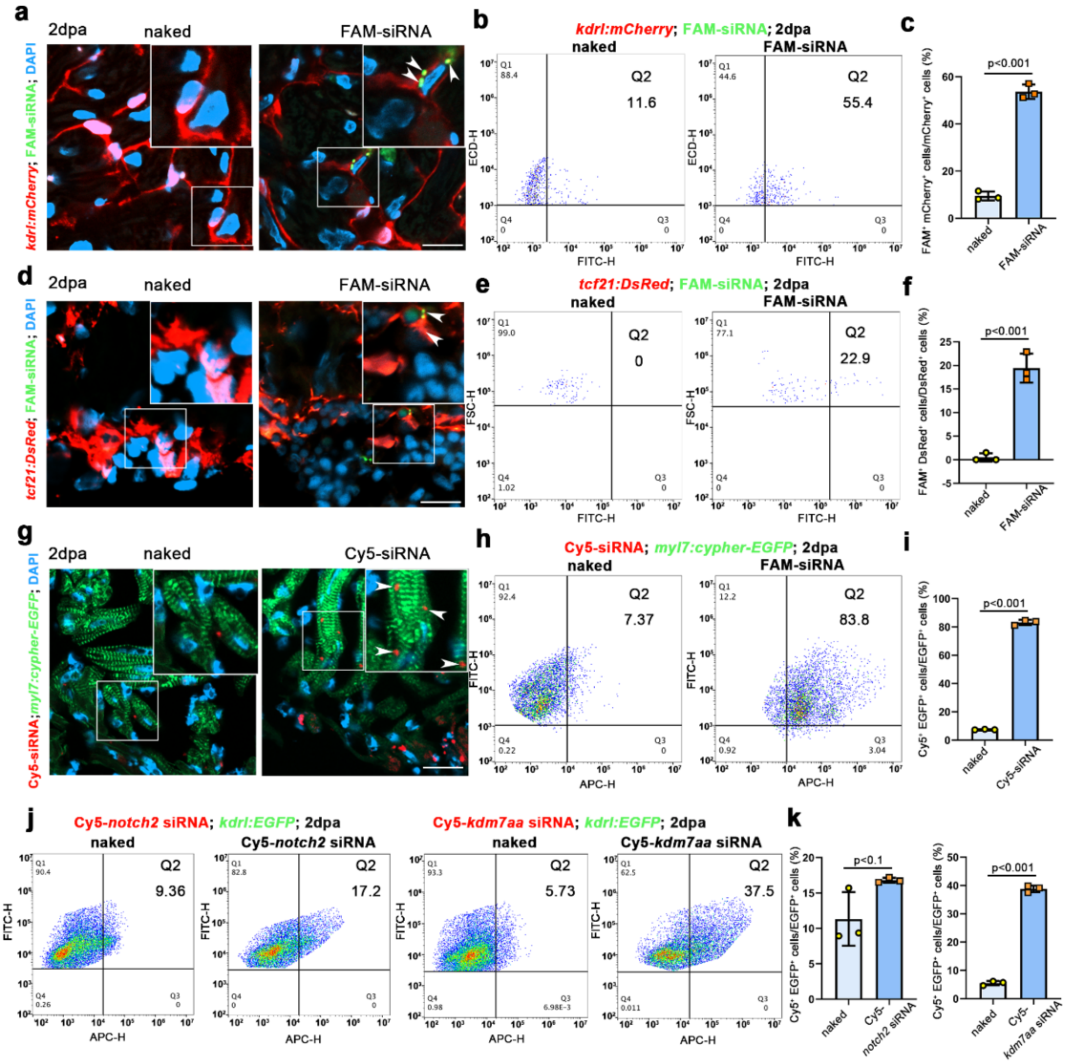

Supplementary Figure 7. Nanoparticle-assisted delivery of siRNA into different cardiac cell types.

a, d, g Confocal images of cryosections of hearts showing that fluorescent encapsulated siRNA (arrowheads) located in the *Tg(kdrl:mCherry)*-labeled endocardium/endothelium (a), *Tg(tcf21:DsRed)*-labeled epicardium (d), and *Tg(myl7:cypher-EGFP)*-labeled cardiomyocytes (g). Note the green FAM-siRNA (a and d) and red Cy5-siRNA (g) signals in the encapsulated siRNA group compared with scarce or no signals in the naked siRNA (naked) group at 2-dpa hearts. The right upper corners presented in a, d and g were high-magnification images of the framed areas. Scale bars, 10 µm. **b-c, e-f, h-i** Flow cytometry (b, e, h) and corresponding quantitative analysis (c, f, i) of FAM-siRNA in the *Tg(kdrl:mCherry)*-labeled endocardium/endothelium (c) and *Tg(tcf21:DsRed)*-labeled epicardium (f), and Cy5-siRNA in the *Tg(myl7:cypher-EGFP)*-labeled cardiomyocytes (i). **j, k** Flow cytometry (j) and corresponding quantitative analysis (k) of Cy5-*notch2* siRNA (left panels of j and k) and Cy5-*kdm7aa* siRNA (right panels of j and k) in the *Tg(kdrl:EGFP)*-labeled endocardium/endothelium. Data were mean fold changes ± s.e.m., n numbers indicated biological replicates, and the range of p-values was shown in the figures, unpaired *t*-test.

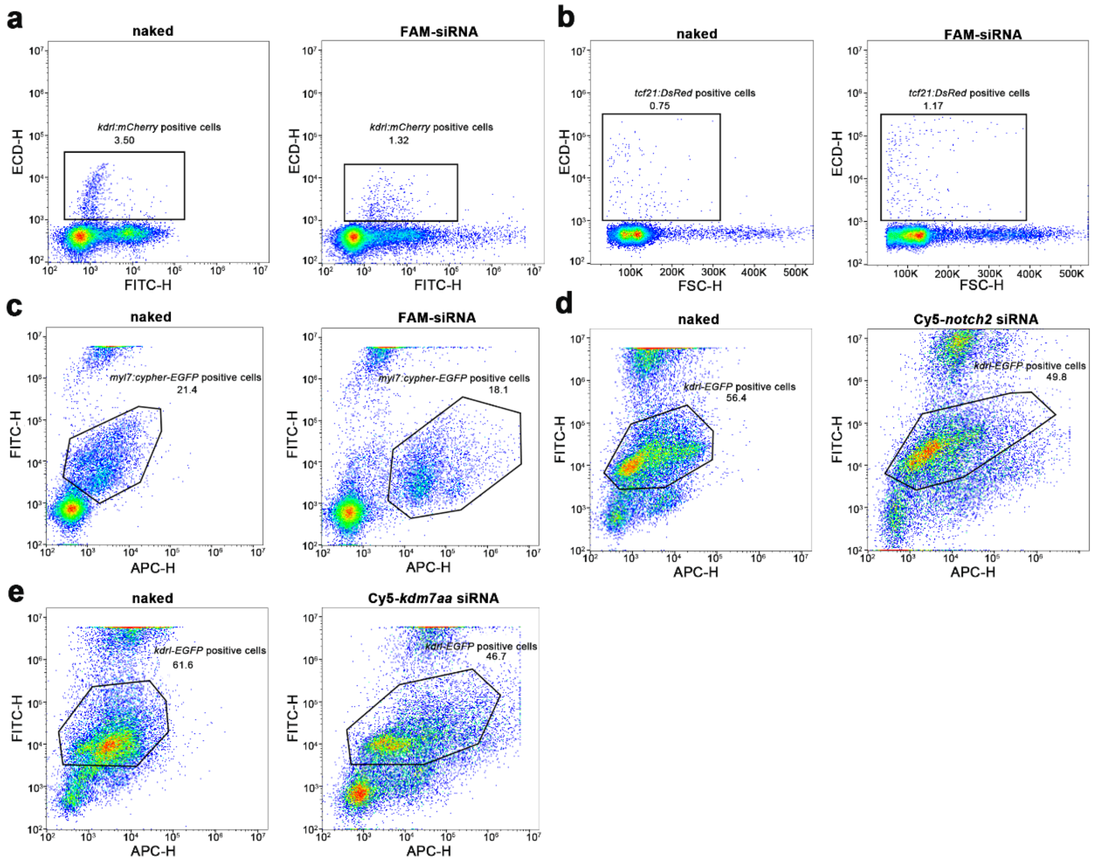

Supplementary Figure 8. The gating strategies used for colocalization analysis of fluorescent siRNA and multitype cell.

a-e *kdrl: mCherry* positive (a, black box), *tcf21:DsRed* positive (b, black box), *myl7: cypher-EGFP* positive (c, black box) and *kdrl:EGFP* positive (d and e, black box) cells were encircled for further FAM^+ $mCherry^+$/$mCherry^+$, FAM^+ $DsRed^+$/$DsRed^+$, and $Cy5^+$ $EGFP^+$/$EGFP^+$ ratio analysis correspond to Supplementary Figure 7b,e,h,j. Data represented one of three experiments.

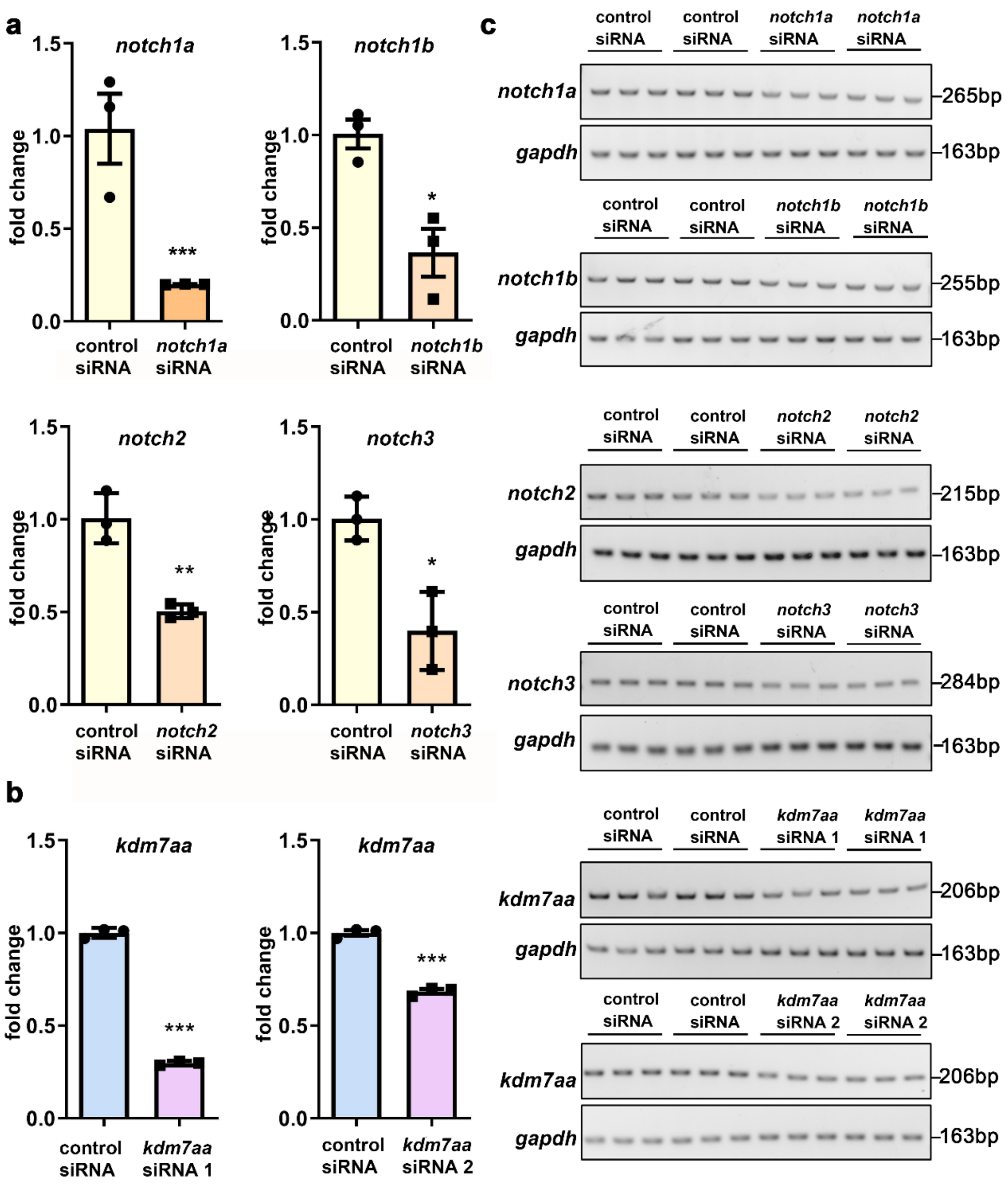

Supplementary Figure 9. Validation of nanoparticle-mediated siRNA knockdown of targeted genes.

a, b Quantitative PCR showing that nanoparticle-mediated *notch1a*, *notch1b*, *notch2*, and *notch3* (a), and *kdm7aa* siRNA (b) decreased the RNA level of each gene in wild-type hearts (n numbers indicated technical replicates; *p <0.05, **p<0.01, ***p<0.005; data were the mean ± s.e.m; unpaired *t*-test). **c** DNA gels with the *gapdh* positive control showing qPCR products of nanoparticle-mediated *notch1a*, *notch1b*, *notch2*, *notch3*, and *kdm7aa* knock down. Gels presented in each panel derived from the same experiment and were processed in parallel.

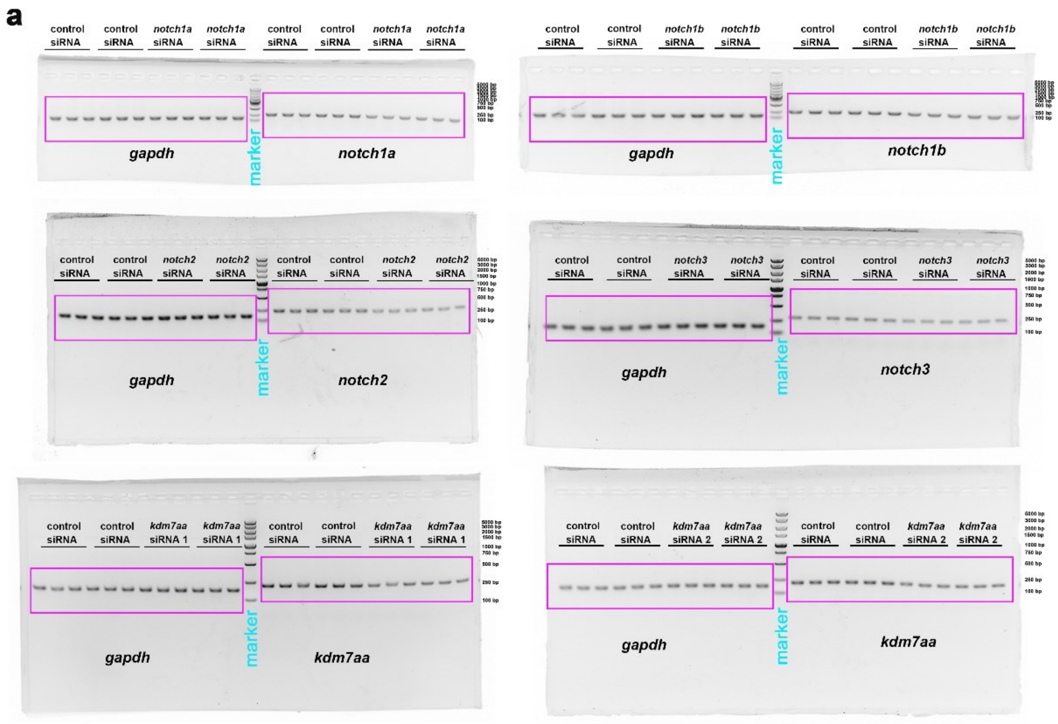

Supplementary Figure 10. Unprocessed gels presented in the manuscript. a The source gel images for Supplementary Figure 9c. Each gel derived from the same experiment and was processed in parallel.

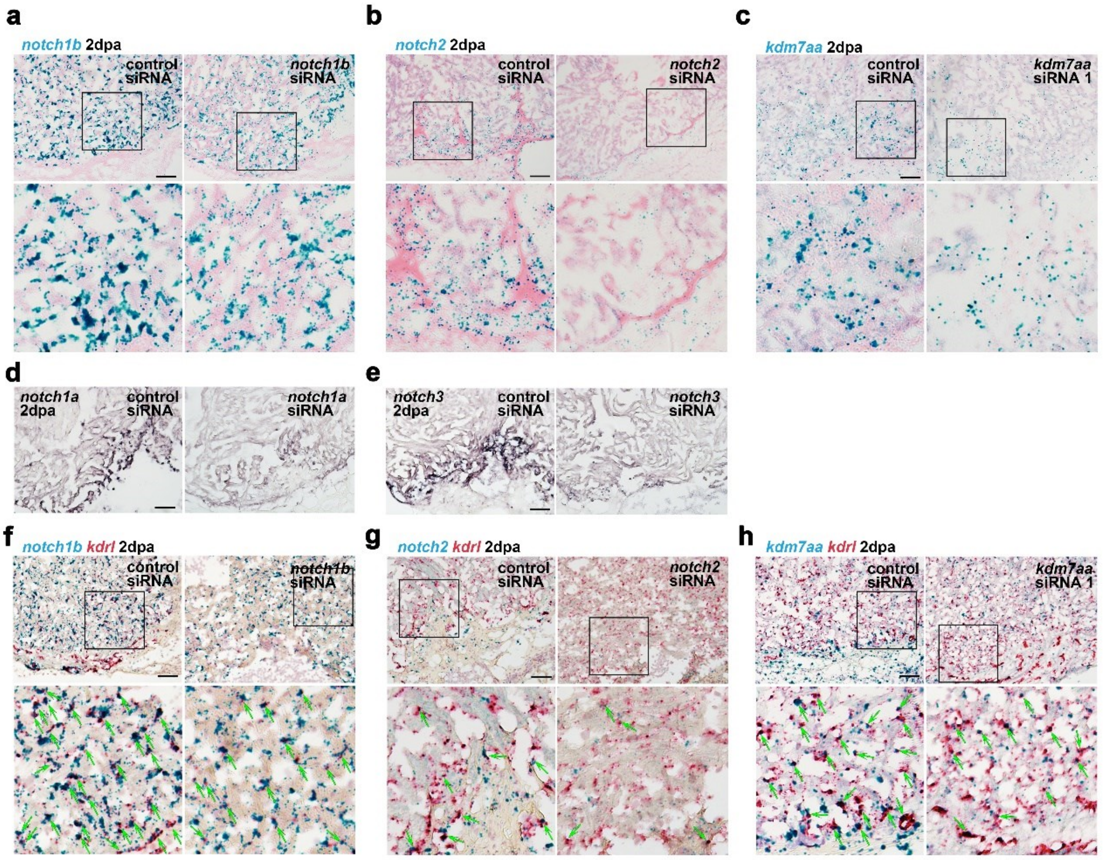

Supplementary Figure 11. Assessment of nanoparticle-mediated siRNA knockdown of targeted genes.

a, b, c RNAscope analysis of *notch1b*, *notch2* and *kdm7aa* probes in frozen sections of 2-dpa hearts injected with encapsulated control siRNA, *notch1b* siRNA, or *kdm7aa* siRNA. The high-magnification images of the framed areas were presented in the corresponding images below. Note the corresponding gene expression signals decreased in the encapsulated *notch1b* siRNA, *notch2* siRNA or *kdm7aa* siRNA groups compared to the encapsulated control siRNA groups. **d, e** Representative images of RNA *in situ* hybridization with *notch1a* probes (d), and *notch3* probes (e) on frozen sections of 2-dpa hearts injected with encapsulated control siRNA, *notch1a* siRNA, or *notch3* siRNA. **f, g, h** RNAscope *in situ* hybridization of 2-dpa heart sections from encapsulated control siRNA, *notch1b* siRNA and *kdm7aa* siRNA groups, using *kdrl* (endothelial cell marker) probes to co-stain with *notch1b* probes (f), *notch2* probes (g) or *kdm7aa* probes (h). The high-magnification images of the framed area were presented below the corresponding images (green arrowheads, double-positive signals for *kdrl*- and *notch1b*-positive endothelium, *kdrl*- and *notch2*-positive endothelium, or *kdrl*- and *kdm7aa*-positive endothelium). Scale bars, 50 μm.

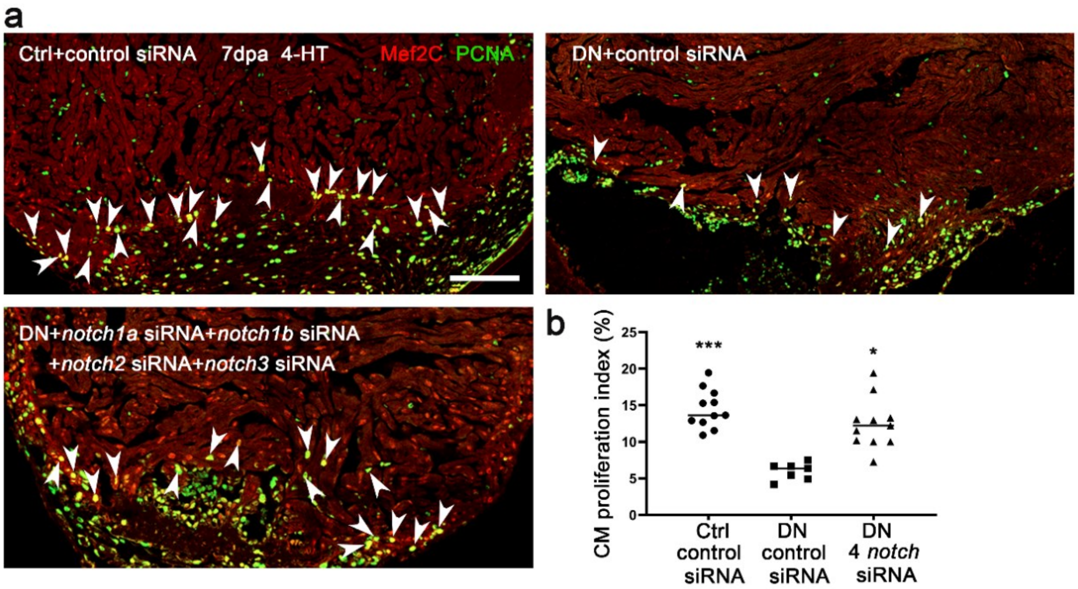

Supplementary Figure 12. Cardiomyocyte-proliferation index in DN-xBrg1 groups is partially rescued by inhibition of Notch signaling.

a Representative image of immunostaining showing that $PCNA^{+}/Mef2C^{+}$ proliferating cardiomyocytes decreased at 7 dpa in DN-xBrg1 hearts (DN) treated with control siRNA (compared with control sibling heats (Ctrl) with control siRNA injection), which were partially rescued by 4 *notch* siRNAs treatment in the presence of 4-HT. Scale bar, 100 µm. **b** Statistics of panel a (data were the mean ± s.e.m.; *$p < 0.05$; ***$p < 0.005$; one-way analysis of variance followed by multiple comparison test).

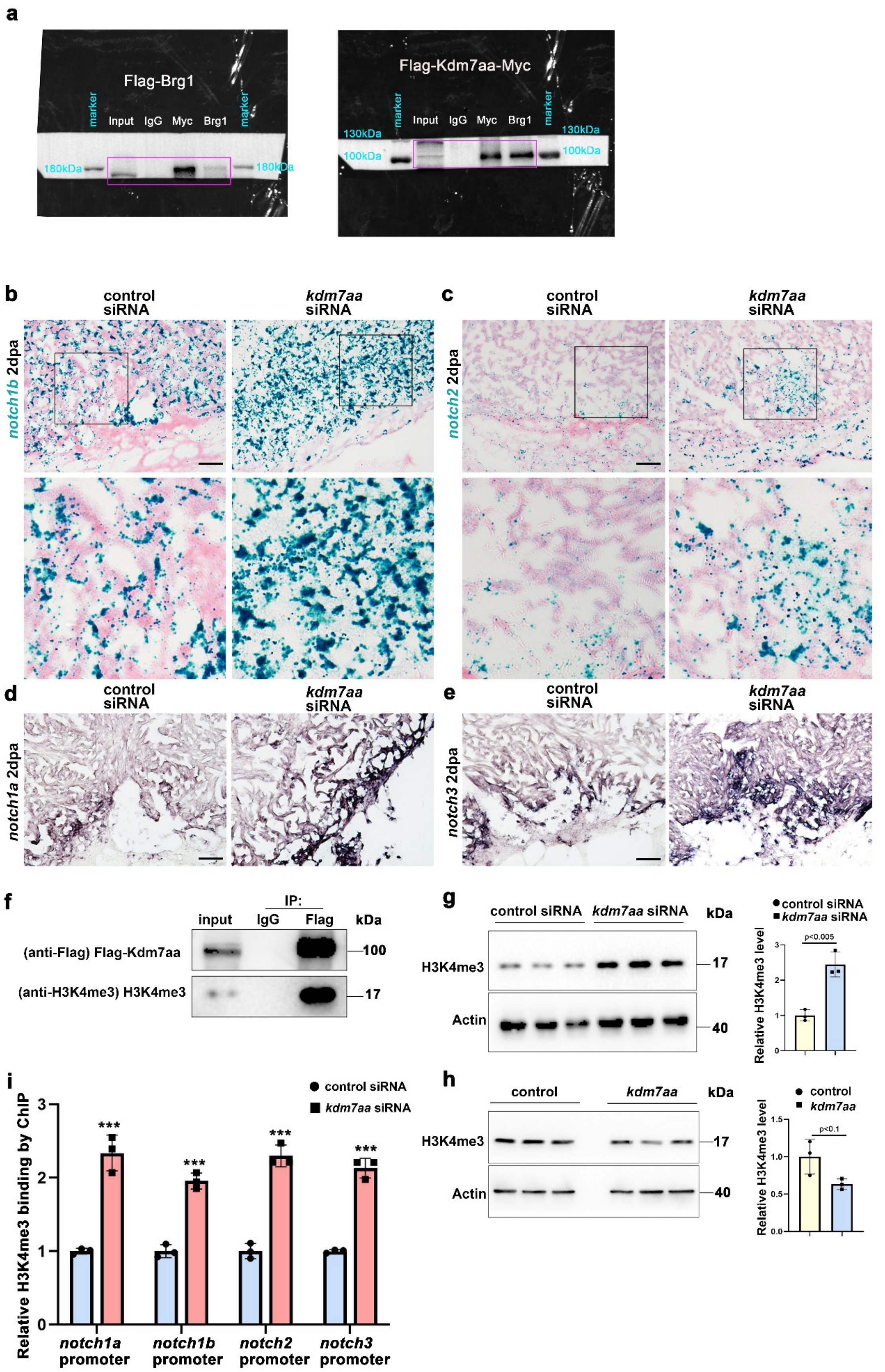

Supplementary Figure 13. Kdm7aa regulates the levels of H3K4me3 modifications at Notch receptors promoters.

a The uncropped blots images for Figure 5e. **b, c** RNAscope analysis of *notch1b* probes (b) and *notch2* probes (c) in frozen sections of 2-dpa hearts injected with encapsulated control siRNA or *kdm7aa* siRNA. The high-magnification images of the framed areas were presented below the corresponding images. Note the *notch1b* and *notch2* signals increased in encapsulated *kdm7aa* siRNA groups compared to the encapsulated control siRNA groups. **d, e** Representative images of RNA *in situ* hybridization with *notch1a* probes (d), and *notch3* probes (e) on frozen sections of 2-dpa hearts injected with encapsulated control siRNA or *kdm7aa* siRNA. **f** Immunoprecipitation assay with anti-Flag antibody revealed that Kdm7aa interacted with H3K4me3 modified histones. Inputs used as loading control and IgG used as negative control. The *Flag-kdm7aa* was overexpressed in 293T cells. **g** Western blot showing elevated H3K4me3 modified proteins in zebrafish hearts treated with *kdm7aa* siRNA. The quantitative analysis was presented in the right side of the panel (n numbers indicated biological replicates, the range of p-values was shown in the figures, unpaired *t*-test). **h** Western blot showing that overexpression of *kdm7aa* decreased H3K4me3 modified proteins in 293T cells. The quantitative analysis was presented in the right side of the panel (n numbers indicated biological replicates, the range of p-values was shown in the figures, unpaired *t*-test.). **i** Anti-H3K4me3 ChIP and quantitative PCR showing elevated bindings to the *notch1a*, *notch1b*, *notch2* and *notch3* promoter regions in 3-dpa hearts injected with *kdm7aa* siRNA compared with control siRNA. Data represented one of three independent experiments and n numbers indicated technical replicates. Data were the mean fold changes ± s.e.m.; ***p <0.005, unpaired *t*-test. Blots from each panel were derived from the same experiment and that they were processed in parallel.

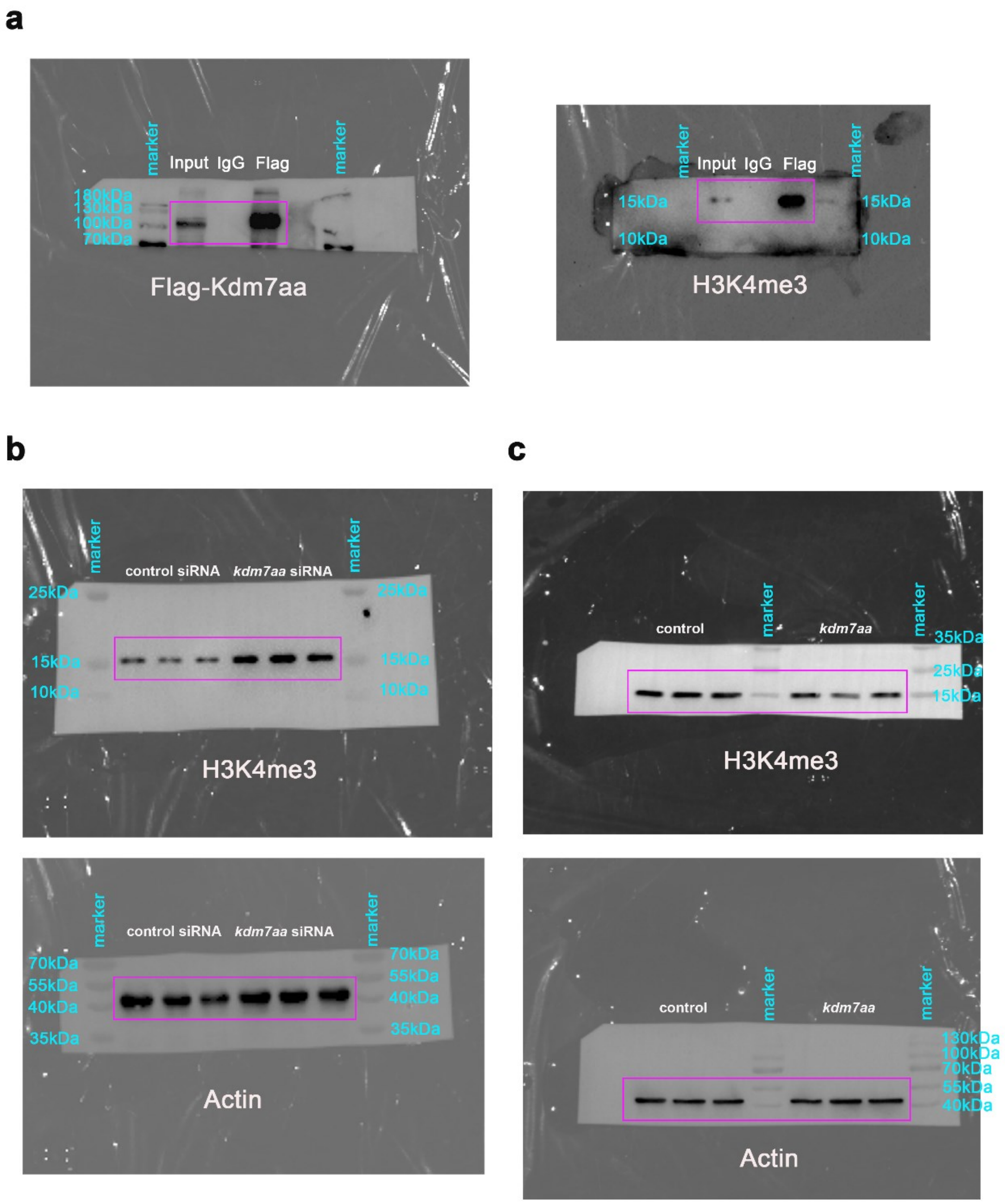

Supplementary Figure 14. Unprocessed images of blots. a The uncropped blots images for Supplementary Figure 13f. **b** The source blot images for Supplementary Figure 13g. **c** The source data for Supplementary Figure 13h. Blots from each panel were derived from the same experiment and that they were processed in parallel.

Supplementary Table 1 List of primer sequences used for Quantitative RT-PCR analysis

**Primer Names**	**Usage**	**Sequences**
notch1a-RT-F	qPCR	CGGCATCAACACCTACAACTG
notch1a-RT-R	qPCR	TGGACACTCGCAGAAGAAGG
notch1b-RT-F	qPCR	AGTGGACGCAGCAGCATT
notch1b-RT-R	qPCR	GGTCTGTCTGGTTGTGAAGGT
notch3-RT-F	qPCR	GGATAACACAGGTCGCTCAC
notch3-RT-R	qPCR	CACCATTCTTCAACAAGGCAAT
notch2-RT-F	qPCR	AACGCAAGCACGGCACTCTG
notch2-RT-R	qPCR	CCTGTCCACTCCATCCACTCCA
dll4-RT-F	qPCR	GGTGGACTGTTCTGTGACCAAGATT
dll4-RT-R	qPCR	CGCAGGTGAGCAGACTGTGTTC
kdm1a RT-F	qPCR	TCCATACAACAGTGATGCCGTCCT
kdm1a RT-R	qPCR	ACTCGTCCACCAACTCGATCTCTT
kdm3b RT-F	qPCR	GCAAGAGCAGTTCTTCAGCACTTCA
kdm3b RT-R	qPCR	GCCAGAGCCAGAGTTCAGCAGAT
kdm5bb RT-F	qPCR	GAGAGGAGATGGACCAAGATCGC
kdm5bb RT-R	qPCR	GCTCGTGTTGCTAGGCTGAAGT
kdm6ba RT-F	qPCR	TAGAGGAGACGCAAGCTGAACGA
kdm6ba RT-R	qPCR	CGGTGAACTGCTCTGCTGTGT
kdm6a RT-F	qPCR	CTTAGCCAGCATAGACAGCACACT
kdm6a RT-R	qPCR	GCAGCATTCTTCCAGTAGTCTGACT
kdm6bb RT-F	qPCR	AATGTCCTGGAGCCTGTCTGAGAA
kdm6bb RT-R	qPCR	GCTGGTGCTGCTGACTGTAAGG
kdm7aa RT-F	qPCR	GGAGGTGTTGAAGAGACTGGAGGTT
kdm7aa RT-R	qPCR	CGTTGACTGTTGCTGCCACATTAG
kdm8 RT-F	qPCR	CGCTACATTACAGGAACCGAGGAAG
kdm8 RT-R	qPCR	TGCGACTCGTGTGGATACAGACT
kdm7ab RT-F	qPCR	TCTCGGACCAACCACACCTCAC
kdm7ab RT-R	qPCR	TCACTACTACTGCTGCTGCTGCT
brg1-RT-F	qPCR	ACACCAGGAGTATCTCAACAGT
brg1-RT-R	qPCR	TCAGCCATAAGCCTTCTCATTC
gapdh-RT-F	qPCR	GATACACGGAGCACCAGGTT
gapdh-RT-R	qPCR	GCCATCAGGTCACATACACG

Supplementary Table 2 List of oligonucleotides applied in this study

**Primer Names**	**Usage**	**Sequences**
notch1a-probe-F	in situ probe	TAATAATGTGGATGCTGCTGTCGT
notch1a-probe-R	in situ probe	CAGACAAGTTGGAATGTGGAGATG
notch1b-probe-F	in situ probe	GCACAAGACGGAAAGGGAAACTTATT
notch1b-probe-R	in situ probe	AATGTGCCTTCATTTAGCGAATC
notch2-probe-F	in situ probe	AGATGGTTTCACTCCTCTCATGCT
notch2-probe-R	in situ probe	AATCCACAGGAGACATGGTAAC
notch3-probe-F	in situ probe	TCTCTGGTAGCCACACACTCTCAC
notch3-probe-R	in situ probe	CCTGAGATGGGATAGCTTGTGCTT
notch1a-pro-F	qChIP	CTGTGCAACAAGTGACGCTCAAAGCGCAAG
notch1a-pro-R	qChIP	CATCGCTCGCGACGGTGGCACAAGGTAACA
notch1b-pro-F	qChIP	CCAGCCAAACGTACCTTGTGTCAAAGTATTGAG
notch1b-pro-R	qChIP	GCCTGCGTGGACTATCCATAAGAAGGAATGC
notch2-pro-F	qChIP	GCGACTTCAGTGACTGGGACGAAAAGAAGAG
notch2-pro-R	qChIP	GAAGCTGTGTTTTGTCTGTCAGGGTGTCTCG
notch3-pro-F	qChIP	GCCTCAGCAACAAAGAGAAAGTGTCCCCATG
notch3-pro-R	qChIP	GTGAGCATCGCCGCAGAACATTACGCAC
notch1a siRNA	sense	GCAUCUGCAUGCCUGGAUA
notch1a siRNA	antisense	UAUCCAGGCAUGCAGAUGC
notch1b siRNA	sense	GCUGGUGAACUGGUGUAAA
notch1b siRNA	antisense	UUUACACCAGUUCACCAGC
notch2 siRNA	sense	GCGAAUGCCCGCCUGGAUATT
notch2 siRNA	antisense	UAUCCAGGCGGGCAUUCGCTT
notch3 siRNA	sense	GCAUCUGUAUGCCUGGCUA
notch3 siRNA	antisense	UAGCCAGGCAUACAGAUGC
kdm7aa siRNA-1	sense	GCUGCUGAUAUCGAUGUUUTT
kdm7aa siRNA-1	antisense	AAACAUCGAUAUCAGCAGCTT
kdm7aa siRNA-2	sense	GCAGGGAACUACCAUCUUATT
kdm7aa siRNA-2	antisense	UAAGAUGGUAGUUCCCUGCTT
control siRNA	sense	UUCUCCGAACGUGUCACGUTT
control siRNA	antisense	ACGUGACACGUUCGGAGAATT
