# Supplementary material for: Endothelial Brg1 fine-tunes Notch signaling during zebrafish heart regeneration
Source: NPJ Regen Med. 2023 Apr 7;8:21. doi: 10.1038/s41536-023-00293-4 (PMC10082087; doi:10.1038/s41536-023-00293-4)
Supplement: Supplementary file 4 — Reporting Summary [file 41536_2023_293_MOESM4_ESM.pdf]

nature portfolio

Corresponding author(s): Jing-wei Xiong

Last updated by author(s): Mar 7, 2023

Reporting Summary

Nature Portfolio wishes to improve the reproducibility of the work that we publish. This form provides structure for consistency and transparency in reporting. For further information on Nature Portfolio policies, see our Editorial Policies and the Editorial Policy Checklist.

Statistics

For all statistical analyses, confirm that the following items are present in the figure legend, table legend, main text, or Methods section.

n/a	Confirmed	
☐	☒	The exact sample size (*n*) for each experimental group/condition, given as a discrete number and unit of measurement
☐	☒	A statement on whether measurements were taken from distinct samples or whether the same sample was measured repeatedly
☐	☒	The statistical test(s) used AND whether they are one- or two-sided<br>*Only common tests should be described solely by name; describe more complex techniques in the Methods section.*
☒	☐	A description of all covariates tested
☒	☐	A description of any assumptions or corrections, such as tests of normality and adjustment for multiple comparisons
☐	☒	A full description of the statistical parameters including central tendency (e.g. means) or other basic estimates (e.g. regression coefficient) AND variation (e.g. standard deviation) or associated estimates of uncertainty (e.g. confidence intervals)
☐	☒	For null hypothesis testing, the test statistic (e.g. *F*, *t*, *r*) with confidence intervals, effect sizes, degrees of freedom and *P* value noted<br>*Give P values as exact values whenever suitable.*
☒	☐	For Bayesian analysis, information on the choice of priors and Markov chain Monte Carlo settings
☒	☐	For hierarchical and complex designs, identification of the appropriate level for tests and full reporting of outcomes
☒	☐	Estimates of effect sizes (e.g. Cohen's *d*, Pearson's *r*), indicating how they were calculated

Our web collection on statistics for biologists contains articles on many of the points above.

Software and code

Policy information about availability of computer code

Data collection	Chromogenic images were examined under Leica DM5000B microscope and collected using LAS V4.4 Software from Leica Application Suite Software. Immunofluorescence images were captured on Carl Zeiss LSM510/980 confocal microscope and collected using ZEN2010 Imaging. RNA-seq and ChIP-seq data collection have been described in the Methods section.
Data analysis	All statistics were calculated using Statistical Product and Service Solutions (SPSS) Software from IBM or GraphPad Prism Software. The signal intensity or area of images was quantified using MBF ImageJ.

For manuscripts utilizing custom algorithms or software that are central to the research but not yet described in published literature, software must be made available to editors and reviewers. We strongly encourage code deposition in a community repository (e.g. GitHub). See the Nature Portfolio guidelines for submitting code & software for further information.

Data

Policy information about availability of data

All manuscripts must include a data availability statement. This statement should provide the following information, where applicable:

- Accession codes, unique identifiers, or web links for publicly available datasets
- A description of any restrictions on data availability
- For clinical datasets or third party data, please ensure that the statement adheres to our policy

Sequencing data that support the findings of this study have been deposited in GEO with the accession codes GSE200936 and GSE200937. The non-sequencing data and/or materials generated during this study are available from the corresponding author upon request.

Human research participants

Policy information about studies involving human research participants and Sex and Gender in Research.

Reporting on sex and gender	*Use the terms sex (biological attribute) and gender (shaped by social and cultural circumstances) carefully in order to avoid confusing both terms. Indicate if findings apply to only one sex or gender; describe whether sex and gender were considered in study design whether sex and/or gender was determined based on self-reporting or assigned and methods used. Provide in the source data disaggregated sex and gender data where this information has been collected, and consent has been obtained for sharing of individual-level data; provide overall numbers in this Reporting Summary. Please state if this information has not been collected. Report sex- and gender-based analyses where performed, justify reasons for lack of sex- and gender-based analysis.*
Population characteristics	*Describe the covariate-relevant population characteristics of the human research participants (e.g. age, genotypic information, past and current diagnosis and treatment categories). If you filled out the behavioural & social sciences study design questions and have nothing to add here, write "See above."*
Recruitment	*Describe how participants were recruited. Outline any potential self-selection bias or other biases that may be present and how these are likely to impact results.*
Ethics oversight	*Identify the organization(s) that approved the study protocol.*

Note that full information on the approval of the study protocol must also be provided in the manuscript.

Field-specific reporting

Please select the one below that is the best fit for your research. If you are not sure, read the appropriate sections before making your selection.

☒ Life sciences ☐ Behavioural & social sciences ☐ Ecological, evolutionary & environmental sciences

For a reference copy of the document with all sections, see nature.com/documents/nr-reporting-summary-flat.pdf

Life sciences study design

All studies must disclose on these points even when the disclosure is negative.

Sample size	Sample size for studies were based on sample sizes used in similar studies performed in the Zebrafish. For quantitating microscopy images, a minimum of 3 biological replicates for each group were obtained. Details on technical and/or biological replicates for individual experiments are provided in Figure Legends and the Materials and Methods section where appropriate.
Data exclusions	No data were excluded from the analysis.
Replication	Details on technical, biological and experiment replicates for individual experiments are provided in Figure legends and the Methods section.
Randomization	Animals were randomly assigned.
Blinding	The investigators were not blinded to group allocation during data collection and/ or analysis.

Reporting for specific materials, systems and methods

We require information from authors about some types of materials, experimental systems and methods used in many studies. Here, indicate whether each material, system or method listed is relevant to your study. If you are not sure if a list item applies to your research, read the appropriate section before selecting a response.

Materials & experimental systems

n/a	Involved in the study
☐	☒ Antibodies
☐	☒ Eukaryotic cell lines
☒	☐ Palaeontology and archaeology
☐	☒ Animals and other organisms
☒	☐ Clinical data
☒	☐ Dual use research of concern

Methods

n/a	Involved in the study
☐	☒ ChIP-seq
☐	☒ Flow cytometry
☒	☐ MRI-based neuroimaging

Antibodies

Antibodies used	Antibodies used for immunofluorescence staining:<br>Primary antibodies anti-Mef2c (HPA005533; Sigma; 1:200), anti-GFP (A-11122; Invitrogen; 1:500), anti-PCNA (P8825; Sigma; 1:300), anti-myosin heavy-chain monoclonal antibody (14-6503-82; eBioscience; 1:500), anti-DsRed (BE3307; EASYBIO; 1:300) and the Brg1 antibody (1:250), which was raised against a glutathione S-transferase-BRG1 fusion protein (human BRG1 amino-acids 1,086-1,307) (PMID: 8232556, 8895581) and Alexa fluorescent-conjugated secondary antibodies Alexa Fluor 488 goat anti-mouse IgG (A21121; Invitrogen; 1:300), Alexa Fluor 488 goat anti-rabbit IgG (A11034; Invitrogen; 1:300), Alexa Fluor 555 goat anti-mouse IgG (A21424; Invitrogen; 1:300), and Alexa Fluor 555 goat anti-rabbit IgG (A21428; Invitrogen; 1:300).<br>Antibodies used for ChIP, qChIP and Immunoprecipitation (IP):<br>Anti-Brg1 (5 μl for each reaction) and anti-H3K4me3 (Ab8580; Abcam; 5 μl for each reaction) antibodies were used for the ChIP assays. The antibodies for IP were anti-Myc (AT0023; Engibody; 5 μg for each reaction), anti-Flag (F3165; Sigma; 5 μg for each reaction), anti-IgG (PI31160; Thermo Fisher Scientific) and anti-Brg1 (10μl for each reaction). The Brg1 antibody used here was a mouse polyclonal antibody raised against GST tagged zebrafish Brg1 amino acid 1,098-1,286.
Validation	All commercial antibodies validation have been described on the manufacturer's website. The Brg1 antibody, which was raised against a glutathione S-transferase-BRG1 fusion protein (human BRG1 amino-acids 1,086-1,307) (PMID: 8232556, 8895581) has been validated in zebrafish in our previously published paper (PMID: 27929112). The Brg1 mouse polyclonal antibody raised against GST tagged zebrafish Brg1 amino acid 1,098-1,286 used here has been validated by immunoprecipitation showing that this Brg1 antibody was able to pull down the Flag-Brg1 in 293T cells (Fig. 5e).

Eukaryotic cell lines

Policy information about cell lines and Sex and Gender in Research

Cell line source(s)	The 293T cell lines were obtained from American Type Culture Collection (CRL-1573; ATCC).
Authentication	The cell lines used were not autheticated.
Mycoplasma contamination	The cell lines tested negative for mycoplasma contamination.
Commonly misidentified lines<br>(See ICLAC register)	No commonly misidentified cell lines were used in this study.

Animals and other research organisms

Policy information about studies involving animals; ARRIVE guidelines recommended for reporting animal research, and Sex and Gender in Research

Laboratory animals	Wild-type TU, Tg(kdrl:EGFP), Tg(kdrl:CreER), Tg(fli1:nucEGFP), Tg(tcf21:DsRed), Tg(myl7:cypher-EGFP), Tg(kdrl:mCherry), Tg(ubi:loxp-DsRed-STOP-loxp-dn-xbrg1) and Tg(ubi:loxp-DsRed-STOP-loxp-NICD) transgenic zebrafish lines were used. Wild-type C57BL/6 mice were used.
Wild animals	No.
Reporting on sex	Male or female fish was randomly selected.
Field-collected samples	No.
Ethics oversight	Male and female zebrafish were raised and handled according to a zebrafish protocol (IMM-XiongJW-3) and the mice used in this study were raised and handled according to the animal protocol (IMM-XiongJW-4), both of witch are approved by the Peking University Institutional Animal Care and Use Committee, which is fully accredited by The Association for Assessment and Accreditation of Laboratory Animal Care International.

Note that full information on the approval of the study protocol must also be provided in the manuscript.

ChIP-seq

Data deposition

☒ Confirm that both raw and final processed data have been deposited in a public database such as GEO.

☒ Confirm that you have deposited or provided access to graph files (e.g. BED files) for the called peaks.

Data access links<br>*May remain private before publication.*	Sequencing data have been deposited in GEO under accession code GSE200937, https://www.ncbi.nlm.nih.gov/geo/query/acc.cgi?acc=GSE200937.
Files in database submission	GSM6046887 Zebrafish heart , 7dpa, Ctrl, H3K4me3<br>GSM6046888 Zebrafish heart , 7dpa, Ctrl, input<br>GSM6046889 Zebrafish heart , 7dpa, DN, H3K4me3<br>GSM6046890 Zebrafish heart , 7dpa, DN, input
Genome browser session<br>(e.g. UCSC)	N/A

Methodology

Replicates	One biological sample for each group.
Sequencing depth	The libraries were sequencing with PE150, the raw reads number was between 35-55 million, and the unique mapped ratio was between 80-83%
Antibodies	Anti-H3K4me3 (Ab8580; Abcam) was used.
Peak calling parameters	STAR --runMode alignReads --genomeDir genomeDir --readFilesIn sample_1P.fq.gz sample_2P.fq.gz --readFilesCommand zcat --outFileNamePrefix sampleID --outFilterMismatchNoverLmax 0.2 --outFilterMatchNmin 20 --alignIntronMax 1 --outFilterMultimapNmax 1 and macs2 callpeak -f BAM -q 0.0001 --broad --nomodel --nolambda -t IP.bam -c INPUT.bam -g genome_size --outdir outDir -n group_name
Data quality	Trim reads after a quality drop below a mean of Q15 in a window of 5 nucleotides and filter out the reads with length below 15 nucleotides. Reads were deduplicated using Picard to mitigate PCR artefacts. The same number of reads (17.5 million pairs) were randomly selected from samples of each condition for peak calling. q-value<0.0001 for peak calling step.
Software	Softwares for data analysis were indicated in the Methods section.

Flow Cytometry

Plots

Confirm that:

☒ The axis labels state the marker and fluorochrome used (e.g. CD4-FITC).

☒ The axis scales are clearly visible. Include numbers along axes only for bottom left plot of group (a 'group' is an analysis of identical markers).

☒ All plots are contour plots with outliers or pseudocolor plots.

☒ A numerical value for number of cells or percentage (with statistics) is provided.

Methodology

Sample preparation	About 15 adult zebrafish hearts were isolated and washed in cold PBS with 10 U/ml heparin (H8060; Solarbio). After the atrium and bulbus were removed, the ventricles were carefully cut into small pieces using forceps and collected into 1.5-ml centrifuge tubes containing cold PBS with 5 mM glucose. The sliced tissue was then transferred to a glass tube along with a magnetic stir bar and 1.5 ml digestion buffer in Dulbecco's modified Eagle's medium containing collagenase type II (250 U/ml) (17101015; Gibco), collagenase type IV (300 U/ml) (17104019; Gibco), and DNase I (30 µg/ml) (A3778; AppliChem). The tube was then transferred to a 32°C water bath with stirring and incubated for 1 min. After incubation, the tube was removed from the water bath and left at room temperature until the tissue settled on the bottom. The supernatant was discarded to remove blood cells, followed by washing once with cold PBS. This was followed by a series of digestion steps with 1.5 ml digestion buffer. Each step consisted of 10 min of digestion followed by 3 min of sedimentation. The supernatants were collected in a 15-ml falcon tube containing 2 ml ice-cold PBS. The cell suspensions were centrifuged at 300 g for 5 min at 4°C, and the cell pellets were gently re-suspended in 1 ml PBS. The resuspended cells were strained using a 70-µm cell strainer and kept on ice for FACS or flow cytometry.
Instrument	Cell sorting was carried out on the Beckman Coulter MoFlo XDP and flow cytometry was carried out on the Beckman Cytoflex EX S.
Software	Data analysis was performed by FlowJo.

Cell population abundance	NO post-sort fraction.
Gating strategy	For cell sorting, debris was removed by gating on the main cell population, and then using the Comp-FL1-Log-H/FL2-H gating to obtain kdrl:EGFP positive cells. Then the FL5-H/FL5-W gating was applied to get kdrl:EGFP singlets as shown in Supplementary Figure 3. EGFP positive singlets were selected for the subsequent RNA isolation. For flow cytometry, gating strategies for individual panels were shown in Supplementary Figure 8.

☒ Tick this box to confirm that a figure exemplifying the gating strategy is provided in the Supplementary Information.
